# Synergistic targeting of cancer-related proteins by benzyl isothiocyanate and caffeic acid: MLSD and cytotoxic mechanisms in MCF-7 cells

**DOI:** 10.1007/s13205-025-04469-1

**Published:** 2025-08-11

**Authors:** Muhamad Hatib A. Rahaman, Nur Aliya Atika Azlan, Mahboob Alam, Nurul Huda Abd Kadir

**Affiliations:** 1 Faculty of Science and Marine Environment, Universiti Malaysia Terengganu, 20130 Kuala Terengganu, Terengganu Malaysia; 2 Department of Safety Engineering, Dongguk University WISE, 123 Dongdae-ro, Gyeongju-si, Gyeongsangbuk-do 38066, Republic of Korea

**Keywords:** Combination therapy, Benzyl isothiocyanate, Caffeic acid, Oxidative stress, MAPK pathway, Apoptosis

## Abstract

This study investigated the synergistic cytotoxic effects of benzyl isothiocyanate (BITC) and caffeic acid (CA) on human breast adenocarcinoma (MCF-7) cells. Cell viability assays (MTT) revealed that BITC induces reactive oxygen species (ROS)-mediated cell death, while CA alone exhibited minimal cytotoxicity. Notably, their combination selectively enhanced MCF-7 cell death with limited effects on human fibroblasts. Mechanistic investigations demonstrated MAPK pathway activation and apoptosis induction, supported by altered expression of GST, p38, p-ERK, ERK 1/2, Nrf-2, and Bcl-2 proteins. Fluorometric analysis revealed significant disruption of redox homeostasis, including changes in ROS, glutathione (GSH), and caspase-3/7 activity. Molecular docking studies confirmed stable binding interactions (binding energies: –4.9 to –6.8 kcal/mol) of BITC and CA with key cancer-related proteins (ERK2, p38 MAPK, Bcl-2, Keap1-Nrf2, GST). Furthermore, Multiple Ligand Simultaneous Docking (MLSD) demonstrated the cooperative binding of BITC and CA to shared and distinct residues within the same protein targets, revealing enhanced binding affinity and potential synergistic inhibition of oncogenic pathways. These results highlight the synergistic potential of BITC and CA to modulate the MAPK pathway, disrupt cellular homeostasis, and induce apoptosis, underscoring their promise for combination cancer therapy.

**Supplementary Information:**

The online version contains supplementary material available at 10.1007/s13205-025-04469-1.

## Introduction

Cancer mortality has already surpassed 9 million people in 2018 (Ferlay et al. 2019). Breast cancer is the most common cancer diagnosed among women, and in 2019, it is estimated that breast cancer alone accounts for 30% of all new cancer diagnoses in women (Siegel et al. 2019), which urges cancer researchers to come up with a new strategy to treat cancer. In line with this, we figure out the solution to this almost endless, devastating disease by looking back to the fundamental biological knowledge that is evolution. If we learn from surrounding nature, evolutionary adaptation in plants gives rise to various bioactive compounds that synergistically interact with each other to survive in their current environment and protect themselves from various pathogens. Appreciating synergism as a gift from nature, we transfer the synergism of bioactive compounds biologically available in edible plants as potential bioactive compounds and their combination concept could benefit our health by producing fascinating synergistic effects to combat cancer.

**Fig. 1:**
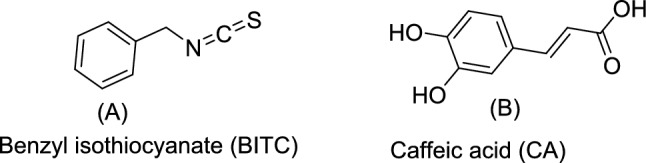
Chemical structure for **A** benzyl isothiocyanate (BITC) and **B** caffeic acid (CA)

The relevance of synergism in cancer treatment is to gain the opportunity to target multiple pathways and mechanisms of action (HemaIswarya and Doble 2006). Drug combination studies have been trending and associated with the emergence of precision medicine in oncology, particularly for immune checkpoint therapy (Katoh 2018; Llovet et al. 2018; Pilié et al. 2017; Duffy 2016), but there has been a lack of studies related to the synergism between dietary approaches and particularly bioactive compounds found in our daily consumption such as benzyl isothiocyanate (BITC) and caffeic acid (CA) (Fig. 1).

Benzyl isothiocyanate (BITC) is a naturally occurring compound found in cruciferous vegetables such as cabbage, mustard, and watercress. Epidemiological studies have shown that the consumption of cruciferous vegetables decreases the risk of various types of cancers (Zhang et al. 2018; Hayes et al. 2008). BITC has been extensively studied for its anticancer effects, demonstrating the ability to suppress tumor initiation, growth, and metastasis across multiple cancer types. Key findings on BITC’s mechanisms include: (1) Induction of Apoptosis and Autophagy: BITC selectively induces apoptosis in cancer cells, driven principally by the generation of reactive oxygen species (ROS) and disruption of mitochondrial membrane potential, leading to mitochondrial pathway-mediated cell death (Nakamura et al. 2018; Lin et al. 2017; Xiao et al. 2008; Xie et al. 2017). BITC also triggers autophagic cell death in breast cancer cells, mediated by FoxO1, and this effect is prominent in multiple breast cancer cell lines. (2) Cell Cycle Arrest: BITC causes cell cycle arrest at different phases, hampering the proliferative capacity of cancer cells. (3) Inhibition of Signaling Pathways: BITC inhibits several key oncogenic pathways, including AKT, STAT3, HDAC, and NF-κB, all of which are involved in tumor growth, survival, and metastasis. (4) Suppression of Angiogenesis and Metastasis: BITC has demonstrated anti-angiogenic activity through suppression of VEGF, HIF-1α, and other angiogenesis-related molecules, correlating with inhibition of STAT3 signaling. (5) Selectivity for Cancer Cells: The compound exhibits preferential cytotoxicity toward cancer cells with limited impact on normal cells. In the context of breast cancer, BITC is effective against both estrogen receptor-positive and triple-negative subtypes. Notably, BITC not only inhibits the growth of breast cancer cells in vitro but also suppresses tumor growth in xenograft models, indicating clinical relevance for breast cancer therapy.

Caffeic acid (CA), a phenolic compound widely found in coffee, vegetables, and fruits (Alam et al. 2022b, 2022a), possesses a range of anticancer mechanisms: (1) Induction of Apoptosis: CA initiates apoptosis by activating caspases, disrupting mitochondrial function, and inducing cell cycle arrest. (2) Anti-proliferative and Anti-angiogenic Effects: CA inhibits tumor cell proliferation and angiogenesis, including downregulation of VEGF expression. (3) Anti-inflammatory and Antioxidant Activities: CA acts as a reactive oxygen species scavenger, decreases oxidative stress, and modulates tumor-promoting inflammatory cytokines (e.g., TNF-α, IL-6), as well as suppressing NF-κB, a central player in inflammation and cancer progression (Espíndola et al. 2019). (4) Immunomodulation: CA enhances natural killer (NK) cell activity and promotes a tumor-suppressing immune microenvironment by affecting macrophage polarization. Numerous studies have suggested that CA reduces viability and possesses an antiproliferative effect on various cancer cells in vitro, including gastric, lung, and breast cancer cells (Chang et al. 2013; Rajendra Prasad et al. 2011). CA’s antiproliferative and apoptosis-inducing effects have been documented in a wide range of tumor models, including breast cancer cells (both ER + and other subtypes).

Several studies have explored the use of BITC and CA in combination with other agents for enhanced therapeutic efficacy. BITC has been combined with chemotherapeutics and targeted agents (such as zoledronic acid and sorafenib) to potentiate anticancer effects, particularly for overcoming drug resistance and inhibiting metastasis in breast cancer models. CA has been formulated in hybrid prodrug systems, exploiting its ROS-generating properties; for example, coupling CA with quinone methide-generating agents amplifies oxidative stress in cancer cells, resulting in synergistic apoptotic cell death. CA has also been evaluated as an adjuvant with standard chemotherapeutics to enhance efficacy and reduce toxicity. While direct combinations of BITC and CA are less frequently reported in the literature, their individual and joint oxidative stress-inducing mechanisms suggest strong synergy potential for targeted cancer cell death.

Both BITC and CA possess multifaceted anticancer activities that target hallmark processes of cancer, such as uncontrolled proliferation, evasion of apoptosis, metastasis, angiogenesis, and immune suppression, key therapeutic targets in breast cancer. Building upon these individual properties and the insights from previous combination studies, this investigation explores the combination of BITC with CA as a novel and innovative model to promote the death of human breast adenocarcinoma (MCF-7) cells (Lim et al. 2024). The rationale for combining BITC and CA in breast cancer therapy includes: (1) Complementary Mechanisms: Both compounds induce oxidative stress and apoptosis via different, yet converging, pathways, increasing the likelihood of redundant or synergistic cytotoxicity toward breast cancer cells. (2) Targeting Multiple Pathways: BITC acts on STAT3, NF-κB, and additional oncogenic targets, while CA further inhibits inflammation and supports immune-mediated tumor suppression. This broad-spectrum targeting counters the plasticity and heterogeneity typical of breast tumors. (3) Selective Toxicity: Both demonstrate limited toxicity to normal cells, suggesting a favorable therapeutic index and potentially fewer side effects than standard chemotherapeutics. (4) Overcoming Resistance and Enhancing Efficacy: The different but complementary mechanisms and molecular targets of BITC and CA may help overcome adaptive resistance mechanisms in breast cancer, particularly in aggressive subtypes where conventional therapies often fail. This combination, therefore, represents a rational strategy to maximize anticancer efficacy by leveraging the individual and overlapping strengths of BITC and CA, supported by a robust molecular rationale and emerging preclinical evidence in the context of breast cancer prevention and therapy. Recent studies have highlighted key molecular targets in breast cancer, providing promising directions for treatment. The mitotic checkpoint regulator BUB1B has emerged as a potential target due to its role in chromosome segregation and its overexpression in breast cancer metastasis (Mishra et al. 2022). Transcriptome analysis has identified BUB1B, AURKA, CCNA2, CCNB2, and PBK as hub genes associated with poor survival outcomes and genomic instability (Mishra et al. 2023b, 2023a). In addition, small molecules that stabilize the G-quadruplex structure in the c-MYC oncogene show potential to inhibit its transcriptional activity in cancer cells (Thumpati et al. 2025). In addition, compounds derived from fungal endophytes are a novel, underexplored source of bioactive metabolites with cytotoxic potential (Prajapati et al. 2025). As dietary bioactive compounds are natural products that occur in food, they have a great potential to gain acceptance by patients and medical practitioners in clinical trials of cancer treatment or/and prevention, albeit approval by regulatory agencies may still be required.

We were adopting synergism in our experimental design by combining BITC with CA as our novel and innovative model of synergism to promote the death of human breast adenocarcinoma (MCF-7) cells. The previous comprehensive review of synergism modeling by Chou (2006) led us to come up with a certain goal in this study, as synergism in cancer treatment increases the efficacy of the therapeutic effect of BITC with the combination of CA by decreasing the dosage but increasing the efficacy while providing selective synergism against cancer cells to avoid toxicity toward non-cancerous cells. Our goal with this study was to shed new light on the BITC-CA synergy. This was accomplished by examining how MCF-7 cells perished following exposure to individual and combined treatments. Analyzing the combination index (CI), the antioxidant system, the MAPK pathway, and the cell death cascade, we were able to gain a better understanding of the synergy.

Furthermore, caffeic acid triggered one PAINS alert (catechol_A) (Baell and Holloway 2010; Daina et al. 2017) due to its catechol group, a functional group known to exhibit redox activity. However, caffeic acid’s biological activity has been well-established in the literature (Chiang et al. 2023; Cortez et al. 2024), particularly its roles in oxidative stress modulation and apoptosis induction. The synergistic effect observed with benzyl isothiocyanate (which has no PAINS alerts) and caffeic acid in this study, combined with molecular docking and mechanistic data, supports the genuine nature of the observed cytotoxicity.

Molecular docking is one of the most widely utilized computational chemistry approaches in the drug discovery process, facilitating the exploration of binding interactions between ligands and the active amino acid residues of target receptors (Kuntz et al. 1982; Wang and Zhu 2016). To support the anticancer potential of the two compounds benzyl isothiocyanate (BITC) and caffeic acid (CA) molecular docking experiments were conducted to gain a better understanding of their binding mechanisms with key receptor proteins. Multiple Ligand Simultaneous Docking (MLSD) provided deeper insights into the cooperative binding behavior of BITC and CA with cancer-related targets. This approach revealed that simultaneous ligand interactions enhance binding stability and suggest a synergistic therapeutic potential (Ischak et al. 2024; Gupta et al. 2022).

## Results

### The combinations of BITC and CA synergistically accelerate the death of MCF-7 compared to human fibroblast cells (CRL-2522)

**Fig. 2:**
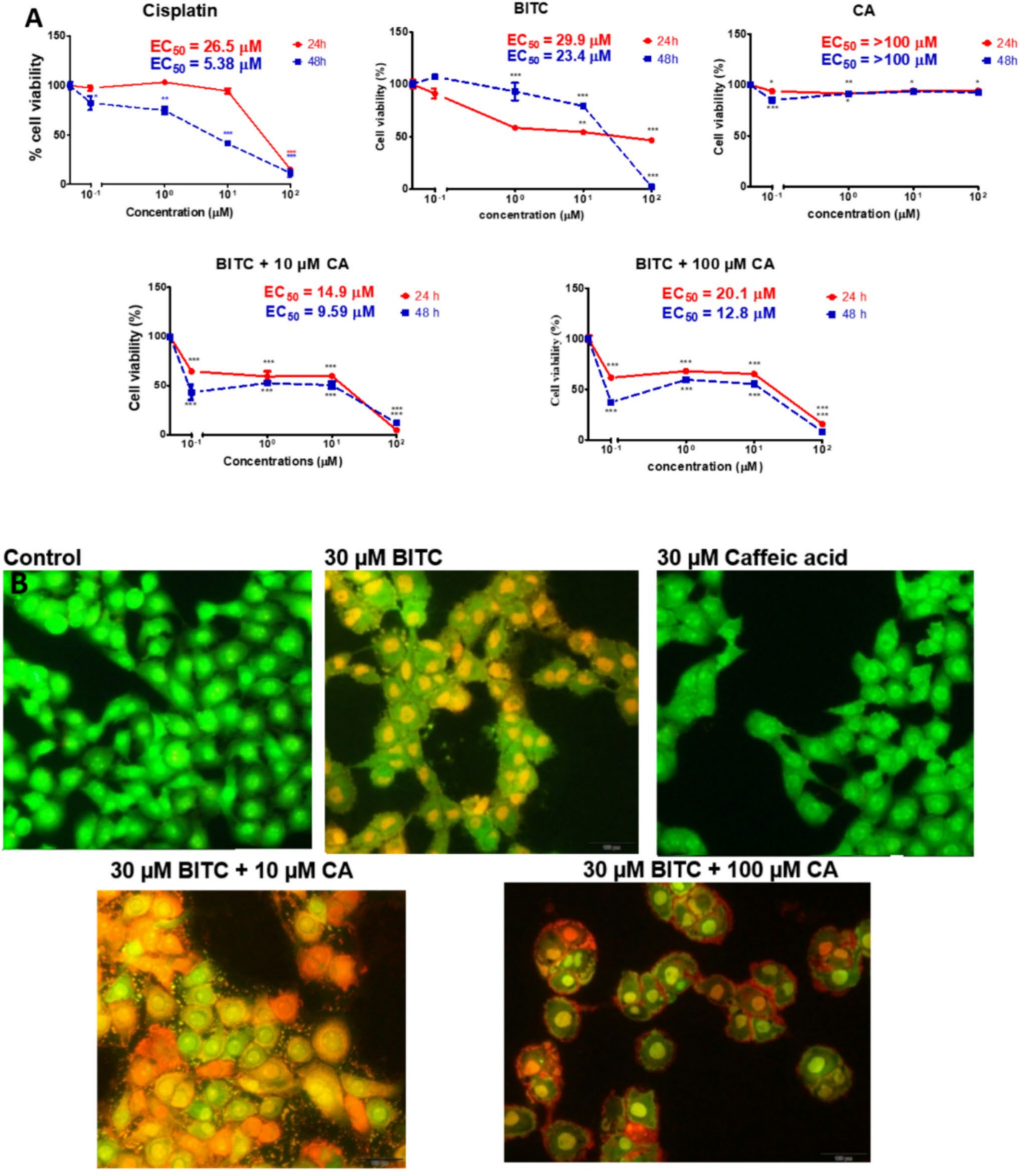

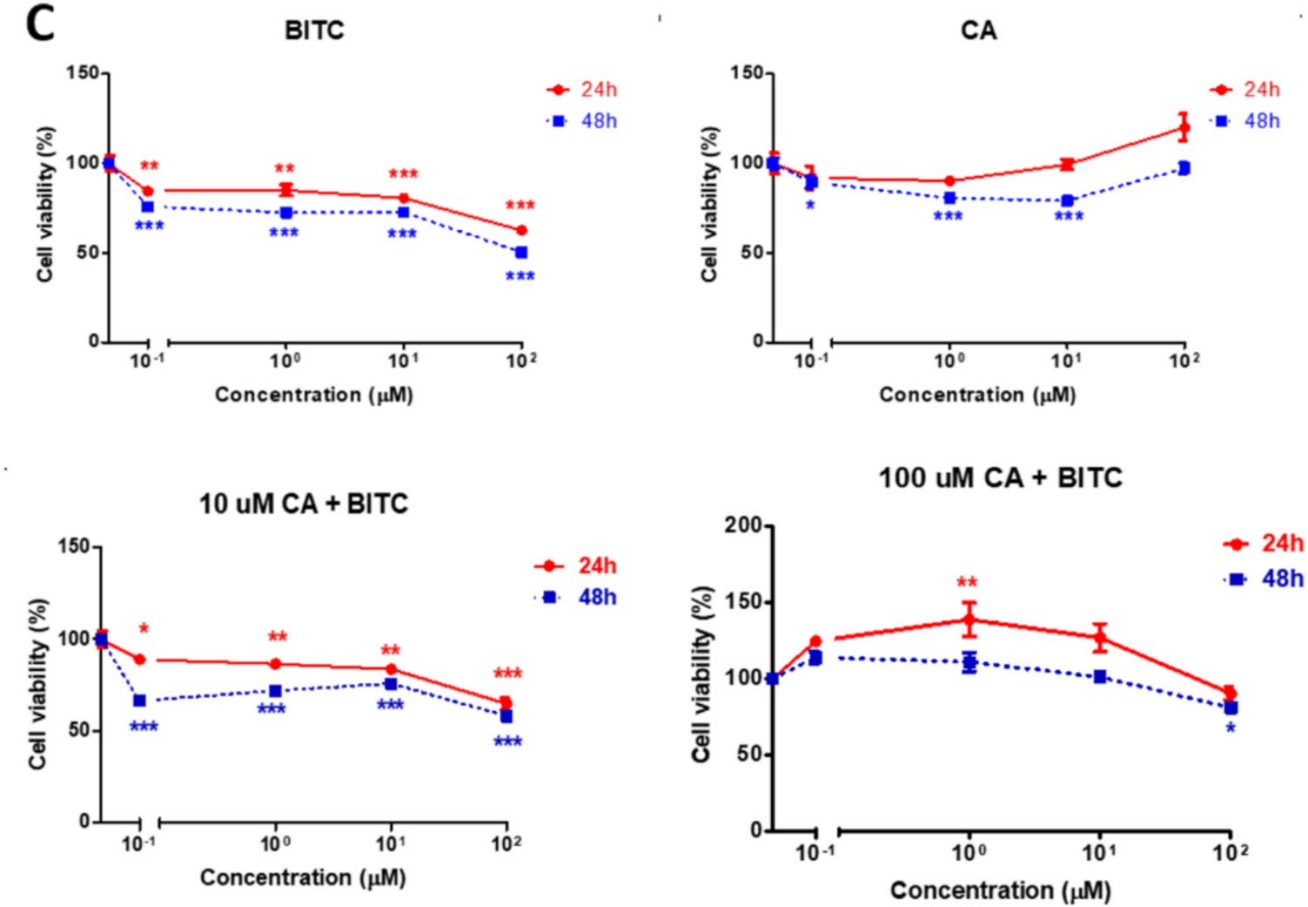
Combination treatment of BITC and CA selectively boosted the death of MCF-7 cells compared to CRL-2522 cells. **A** Results for MTT dye absorbance are shown as inhibition of metabolic activity (percent different from control) and are means ± SEM (n = 4). **B** Morphological changes of the 24 h treated MCF-7 cells can be seen after dual staining of acridine orange and ethidium bromide (AO/EB). The stained MCF-7 cells were characterized as viable (light green), early apoptotic (bright green with condensed chromatin or bright yellow nucleus) late apoptotic (orange nucleus), and nonviable necrotic cells (red) was examined under a fluorescent microscope (X 400 magnification). **C** Effect of treatments on human fibroblast cells CRL-2522 with BITC, CA, and their combinations at 24 and 48 h as assessed by MTT dye absorbance. Results for MTT dye absorbance are shown as inhibition of metabolic activity (percent different from control) and are means ± SEM (n = 4)

BITC inhibited MCF-7 cell survival more effectively than CA in a time- and dose-dependent manner throughout a single compound treatment (Fig. 2A). As a control, MCF-7 cell viability remained unchanged after CA exposure for 24 and 48 h at all dose levels. Fascinatingly, the combination of BITC with 10 µM CA increased the effectiveness by up to 2.01 and 2.44 folds after 24 and 48 h, respectively, compared to a single treatment of BITC to induce MCF-7 cell death. In addition, BITC + 100 µM CA increased the treatment efficacy up to 1.49 and 1.83 folds after 24 and 48 h. Double sequential acridine orange and ethidium bromide staining (AO/EB) was conducted as a reliable morphological assessment to discriminate the modes of cell death(Mironova et al. 2007) (Fig. 2B). Apoptotic bodies can be seen after the exposure of BITC to MCF-7 for 24 h, while CA-treated cells show similar morphology to control except for less proliferation. Combination-treated cells with BITC, and CA show mixed modes of cell death, which are apoptosis and necrosis, that are caused by the acute event of cell death induction created by the increased synergism treatment efficacies. Besides that, disruption of the membrane by the various treatments on MCF-7 cells can be seen with the membrane blebbing on apoptotic cells. None of the treatments on human fibroblast cells (CRL-2522) produced an EC_50_ value, which indicates the treatment is not reaching the standard toxicity to induce cell death (Fig. 2C). BITC did reduce the viability of human fibroblast cells, but not to 50% after maximum concentration exposure, and CA had only a marginal effect on cell viability. The combination treatment, like single treatments, has marginal toxicity, and interestingly, the combination of 1 µM BITC with 100 µM CA causes proliferation in human fibroblast cells of up to 39% at 24 h.

### Synergism quality of BITC and CA combinations treatment boosted MCF-7 cell death

**Fig. 3:**
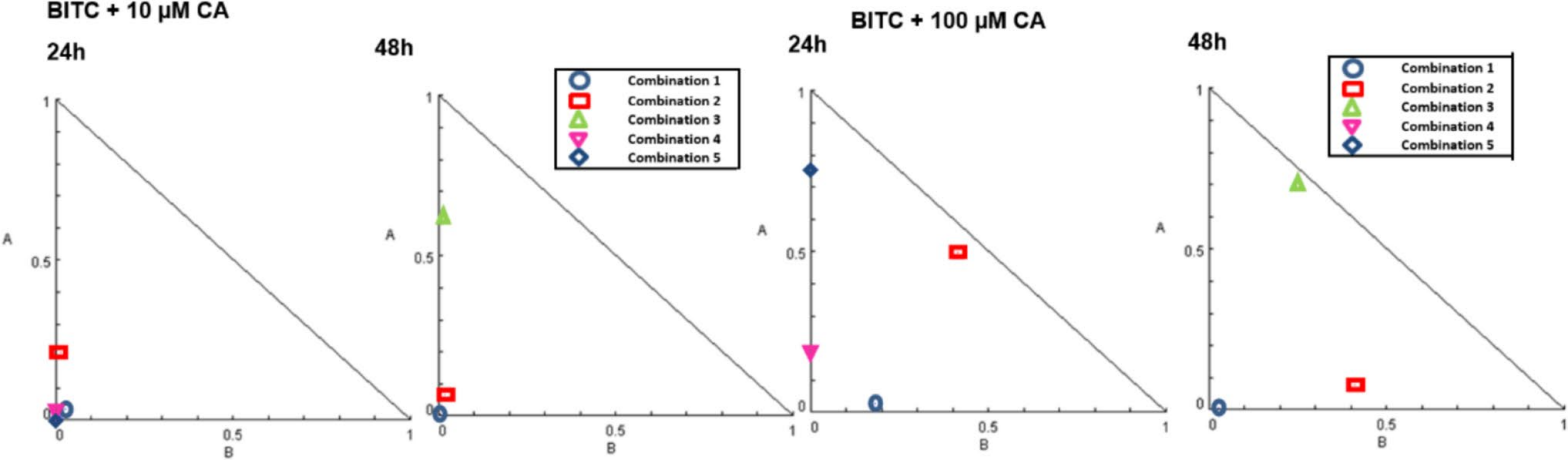
The synergism quality of BITC and CA combinations boosted the death of MCF-7 cells. **A** Normalized isobolograms of synergism analysis of BITC + 10 µM CA and BITC + 100 µM CA after 24 and 48 h treatments are represented in the normalized isobologram plot of MCF-7 cells. The end of CI values for synergism ranging from 0 to 1 and for antagonism it starts from 1 to infinity

Isobolograms were generated based on the CI value of BITC and CA combination exposure on MCF-7 cells as shown in Fig. 3. For 24 h treatment of BITC + 10 µM CA, combinations 1, 2, 4, and 5 show synergism CI values, whereas for 48 h, combinations 1, 2, and 3 show synergistic effects CI values (Table 1). Interestingly, when the concentration of CA was increased from 10 to 100 µM, the same sets of combinations possessed synergism indicated CI values. Impressively, five combinations in BITC + 10 µM CA show very strong synergism CI values (CI < 0.1) which are combinations 1, 4, 5 for 24 h and combinations 1, 2 for 48 h. Moreover, one combination of BITC + 100 µM CA (0.1 µM BITC + 100 µM CA) shows a very strong synergism CI value at 48 h. Combination 2 (24 h) for BITC + 10 CA along with combinations of 1 and 4 at 48 h shows strong synergism CI values (CI 0.1–0.3). Meanwhile, combinations 2 (24 h) and 3 (48 h) for BITC + 100 µM CA show nearly additive CI values (CI = 0.90 – 1.10). The dose-reduction index (DRI) indicates the potential fold of BITC and CA doses in a synergistic range that can be reduced while maintaining similar booster efficacy to kill MCF-7 cells. As shown in Table 1, combination 5 (24 h) of BITC + 10 µM CA has the highest value of DRI, which suggests 231-folds of BITC doses could be reduced in the further study of BITC + CA combination treatment.

**Table 1:** Combination index (CI), dose reduction index (DRI), and viability percentage of MCF-7 cells at 24 and 48 h treated with BITC and CA combinations. CI < 1 indicates a synergistic effect, CI 1 indicates additive effects and CI > 1 indicates an antagonistic effect as described by Chou and Talalay (1983). Chou (1991) had refined CI values as very strong synergism (< 0.1), strong synergism (0.1–0.3), synergism (0.3–0.7), moderate synergism (0.7–0.85), slight synergism (0.85–0.90), nearly additive (0.90–1.10), slight antagonism (1.10–1.20), moderate antagonism (1.20–1.45), antagonism (1.45–3.3), strong antagonism (3.3–10) and very strong antagonism (> 10)

Combination no	Combination (µM)		Cell viability (%) ± SD	CI value	DRI (BITC)	DRI (CA)
	BITC dosage	CA dosage				
1 (24 h)	0.1	10	64.5675 ± 4.5771	0.0618	28.489	37.463
2 (24 h)	1.0	10	59.3790 ± 10.4550	0.2257	4.728	70.679
4 (24 h)	100.0	10	4.7508 ± 1.3635	0.0090	111.039	1,185,837
5 (24 h)	1000.0	10	1.3132 ± 0.5614	0.0043	231.231	5.329E7
1 (48 h)	0.1	10	43.2199 ± 15.4804	0.0108	193.470	176.848
2 (48 h)	1.0	10	52.5478 ± 4.6973	0.0833	15.224	56.838
3 (48 h)	10.0	10	50.5375 ± 6.8019	0.6377	1.603	72.526
1 (24 h)	0.1	100	61.5087 ± 3.5775	0.2088	38.525	5.468
2 (24 h)	1.0	100	68.0049 ± 3.5110	0.9158	2.000	2.405
4 (24 h)	100.0	100	15.7731 ± 1.7847	0.1886	5.312	2626.32
5 (24 h)	1000.0	100	11.1742 ± 0.9067	0.7547	1.325	8259.77
1 (48 h)	0.1	100	37.2460 ± 1.0415	0.0310	226.821	37.563
2 (48 h)	1.0	100	59.4785 ± 3.7948	0.4916	12.715	2.422
3 (48 h)	10.0	100	55.3064 ± 3.5906	0.9514	1.418	4.061

### Regulation of oxidative stress and activation of MAPK pathway mediated MCF-7 cell death

**Fig. 4:**
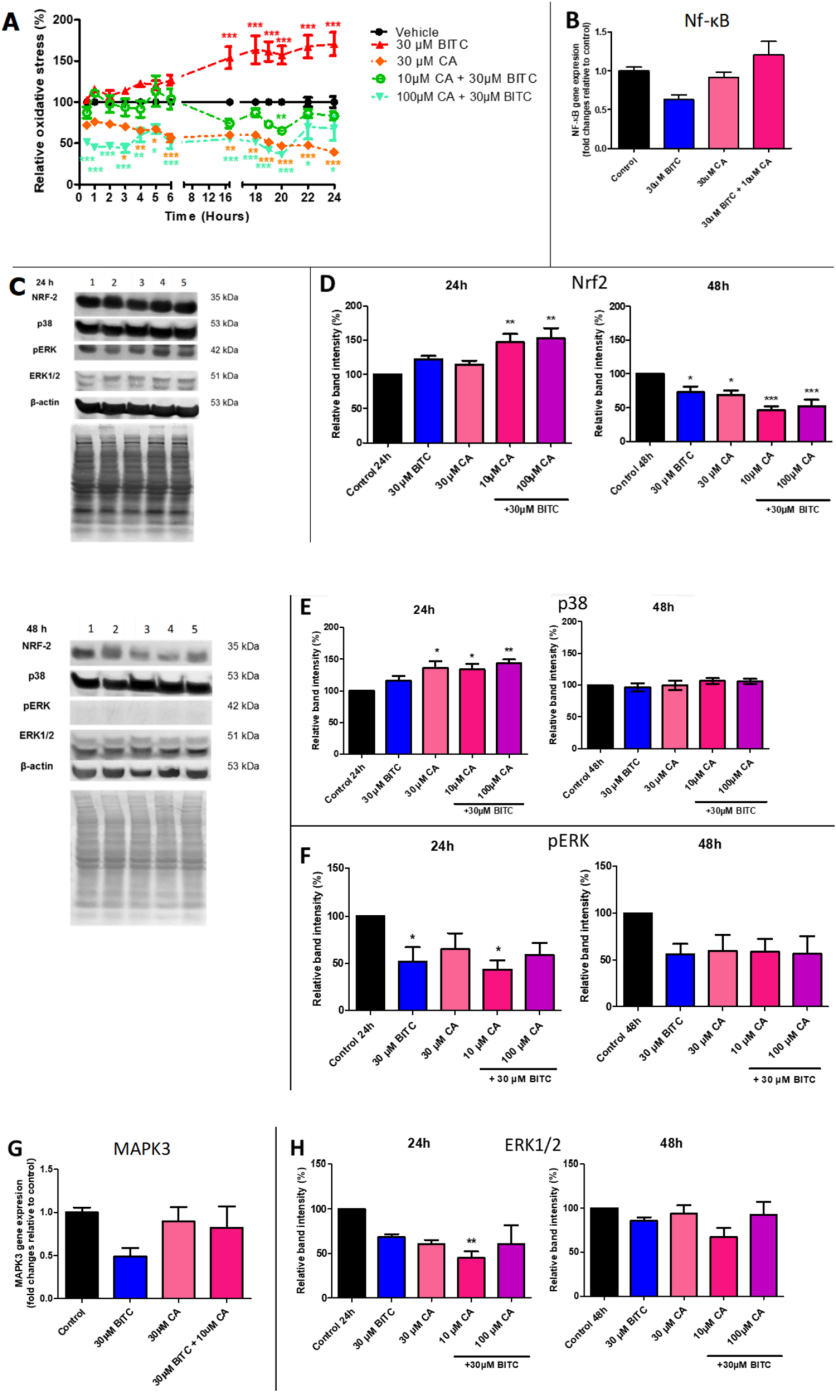
BITC + CA combination treatment regulated oxidative stress and activated the MAPK pathway of MCF-7 cells. **A** Reactive oxygen species (ROS) percentage level of MCF-7 cells with BITC, CA, and BITC + CA at 30 min, 1 h, 2 h, 3 h, 4 h, 5 h, 6 h, 17 h, 18 h, 19 h, 20 h, 22 h and 24 h as assessed by DCFDA dye fluorometrically at excitation of 485 nm and emission of 530 nm. BITC-mediated oxidation of H2DCFDA to fluorescent 2,7-dichlorofluorescein as the result of ROS generation. Each value is the mean ± SEM (n = 3). Two-way ANOVA (Bonferroni post-test) analysis was performed. *P < 0.05, **P < 0.01, ***P < 0.001. **B** Relative gene expression NF-κB analysis by qPCR after 24 h treatment of MCF-7 cells. **C** Expression analysis of MCF-7 cells protein after 24 and 48 h of the respective treatments; 1) control (0.01% DMSO); 2) 30 µM BITC; 3) 30 µM CA; 4) 10 µM CA + 30 µM BITC; 5) 100 µM CA + 30 µM BITC. MCF-7 cells were analyzed for Nrf2, p38, pERK, and ERK1/2 expression by western blot. Loading controls were labeled as housekeeping protein β-actin and the protein bands were normalized by stained-blotted protein on the PVDF membrane. **D** Percentage of relative band intensity of NRF-2 protein expression for untreated and treated MCF-7 cells after 24 and 48 h. The analysis was done by using Image Lab software version 6.0. **E** Expression of p38 protein of MCF-7 cells after 24 and 48 h. **F** Protein expression intensities of p-ERK protein of MCF-7 cells. **G** Relative gene expression MAPK3 for untreated and treated MCF-7 cells after 24 h by qPCR. **H** Percentage of relative band intensity of ERK1/2 protein expression for untreated and treated MCF-7 cells after 24 and 48 h

In comparison to the control, BITC elevated ROS levels and steadily accelerated ROS production from one to twenty-four hours (Fig. 4A). BITC mediated oxidation of H2DCFDA to fluorescent 2,7-dichlorofluorescein. In contrast, CA decreased ROS levels from the first (30 min) until the last time point (24 h). Regardless of whether BITC is present in combination treatments, the higher dose of CA causes more depletion of ROS levels compared to control, in time, and dose-dependent. For example, BITC in combination with the highest dose of CA (100 µM) marked as the lowest ROS level among all treatments but still boosted the death of MCF-7 cells. 0.0003% hydrogen peroxide served as a positive control (data not shown). Furthermore, the expression of Nrf2 protein was up-regulated after 24 h for all treatments compared to the control. Single treatments of BITC and CA increased Nrf2 expression but not significantly, while both of the combination treatments significantly up-regulated Nrf2 after 24 h on MCF-7 cells (Fig. 4D). In contrast with the intense Nrf2 expression trend upon 24 h treatment, Nrf2 was significantly down-regulated after 48 h for all treatments. Besides that, treatment of 24 h of 30 µM BITC induced a marginal decrease of NF-κB gene expression and almost no changes for CA and combination treatments compared to control (Fig. 4B). In this study, BITC affected MAPK signaling-associated proteins such as p38, ERK, ERK1/2 (Fig. 4C) and also the MAPK3 gene (Fig. 4G). At 24 h treatment, p38 MAPK was up-regulated for all treatments with the significant regulation of the single treatment of CA and both of the BITC + CA combinations treatment (Fig. 4E). Interestingly, all treatments were not regulated by p38 MAPK as the control and were insignificant after 48 h of treatments. From the data in Fig. 3F, all treatments down-regulated phosphorylated-ERK (p-ERK) protein expression after 24 and 48 h. Single treatment of BITC and combinations of BITC + 10 µM CA significantly reduced ERK phosphorylation activity by 51.69% and 43.51%, respectively, at 24 h. The p-ERK expression was decreased for all treatments but not significant at 48 h. Insignificant down-regulation of ERK1/2 MAPK can be seen for all treatments in 24 h except for the combination treatment BITC + 10 µM CA, with 45.13% ERK1/2 significant inhibition compared to control (Fig. 4H). Inconsistent ERK1/2 expressions at 48 h were not significant and did not show any remarkable trend. Meanwhile, the gene expression of MAPK3 is not significant for all treatments (Fig. 4G). BITC treatment shows down-regulation of the MAPK3 gene after 24 h treatment, while combination treatment with CA failed to regulate MAPK3.

### Coordination of Bcl-2, caspase 3/7 and phase II of xenobiotic metabolism by the up-regulation of GST revealed the downstream intracellular signaling to cause MCF-7 cells death

**Fig. 5:**
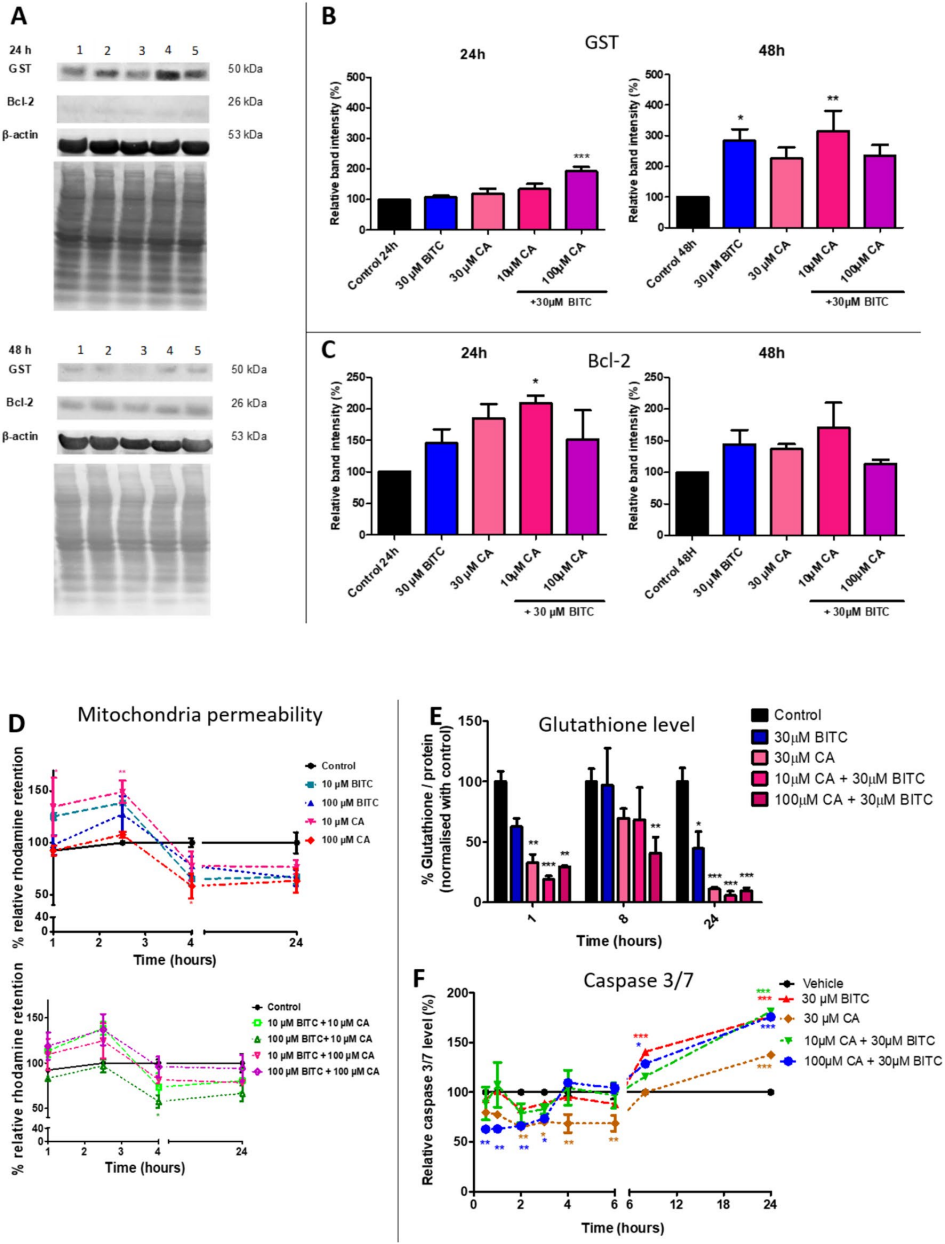
BITC + CA combination treatment coordinated downstream signaling of MCF-7 cell death after interrupting the balance of redox homeostasis. **A** Protein expression analysis of MCF-7 cells after 24 and 48 h of the treatments exposure; 1) control (0.01% DMSO); 2) 30 µM BITC; 3) 30 µM CA; 4) 10 µM CA + 30 µM BITC; 5) 100 µM CA + 30 µM BITC. MCF-7 cells were analyzed for GST and Bcl-2 expression by western blot. β-actin was labeled as a loading control and the protein bands were normalized by stained-blotted protein on the PVDF membrane. **B** GST expression of the treated MCF-7 cells after 24 and 48 h. The analysis was done by using Image Lab software version 6.0. **C** Percentage of relative band intensity of Bcl-2 protein expression for untreated and treated MCF-7 cells after 24 and 48 h. **D** Relative Rh123 fluorescent retention percentage of treated MCF-7 cells at 1, 2, 4, and 24 h. Fluorometric analysis of Rh123 was performed to measure the mitochondrial trans-membrane potential (ΔΨ_m_) at excitation of 500 nm and emission of 550 nm. **E** GSH level of MCF-7 cells after treatments with BITC, CA, and their combinations (10 and 100 µM) at 24 h as assessed by OPA fluorescence intensities. Results for GSH levels of the treatments are shown as a percentage of GSH/ total protein content and data are means ± SEM (n = 4). One-way ANOVA (Dunnet test) was performed. **F** Caspase 3/7 level of MCF-7 cells with BITC, CA, and BITC + CA at 30 min, 1, 2, 3, 4, 6, 8, and 24 h as assessed by FLICA dye fluorometrically at excitation of 488 nm and emission of 530 nm. Each value is the mean ± SEM (n = 3). Two-way ANOVA analysis (Bonferroni post-test) was performed. * indicates P < 0.05, **indicates P < 0.01 and *** indicates P < 0.001 significant difference between control and treatment

GST protein expression was significantly increased (92.25% more than control) for the combination treatment of BITC + 100 µM CA at 24 h while all other treatments showed insignificant GST up-regulation (Fig. 5B). Interestingly, all treatments up-regulated GST expression at least onefold more than control at 48 h. BITC and BITC + 10 µM CA significantly upregulated GST by 183 and 214% more than the control, respectively, whereas treatment of CA and BITC + 100 µM CA insignificantly upregulated GST by 125 and 134% more than the control, respectively. Retention of rhodamine-123 (Rh123) is assessed to measure the mitochondrial trans-membrane potential (ΔΨ_m_) that will affect cytochrome c release and cascades of cell death. A lot of noise can be seen at 1 and 2 h of treatments (Fig. 5D). Consecutively, the effect of mitochondrial membrane permeability only becomes readily apparent after 4 h as the relative retention level of Rh123 decreased for all treatments until the end of the time point (24 h) compared to control. Thus, the effects of mitochondrial membrane permeability toward treatments are dose and time-dependent. The treatments of 100 µM CA and 100 µM BITC + 10 µM CA significantly decreased Rh123 fluorescent retention at 4 h, suggesting prominent rupture of the mitochondrial membrane caused further loss of mitochondrial membrane integrity towards the end of the experiment. As Fig. 5E shows, the amount of GSH in MCF-7 cells was significantly depleted after 1 h for treatments of CA, BITC + 10 µM CA, and BITC + 100 µM CA. Meanwhile, a single treatment of BITC depleted GSH but was not statistically significant compared to the control. BITC + 100 µM CA was the only treatment that significantly fluctuates GSH level in MCF-7 cells at 8 h compared to all other treatments. Interestingly, all treatments at 24 h were observed to cause significant depletion of GSH levels, including the most significantly (P < 0.001) depleted GSH level by BITC + 10 µM CA and BITC + 100 µM CA treatments compared to control. GSH levels were significantly depleted after 24 h for all treatments compared to the control. Interestingly, Bcl-2 was up-regulated throughout all treatments at 24 and 48 h but only BITC + 10 µM CA combination treatment at 24 h significantly increased Bcl-2 expression of MCF-7 cells (Fig. 5C). The data shows the increased caspase 3/7 level upon 8 until 24 h for CA, BITC, and BITC + CA combinations treatments (Fig. 5F). The elevation of caspase 3/7 level started late as the caspase 3/7 located at the downstream of the apoptotic pathway. CA produced less caspase 3/7 level as CA is less toxic toward MCF-7 cells compared to BITC and BITC + CA treatments.

### Molecular docking analysis

**Fig. 6:**
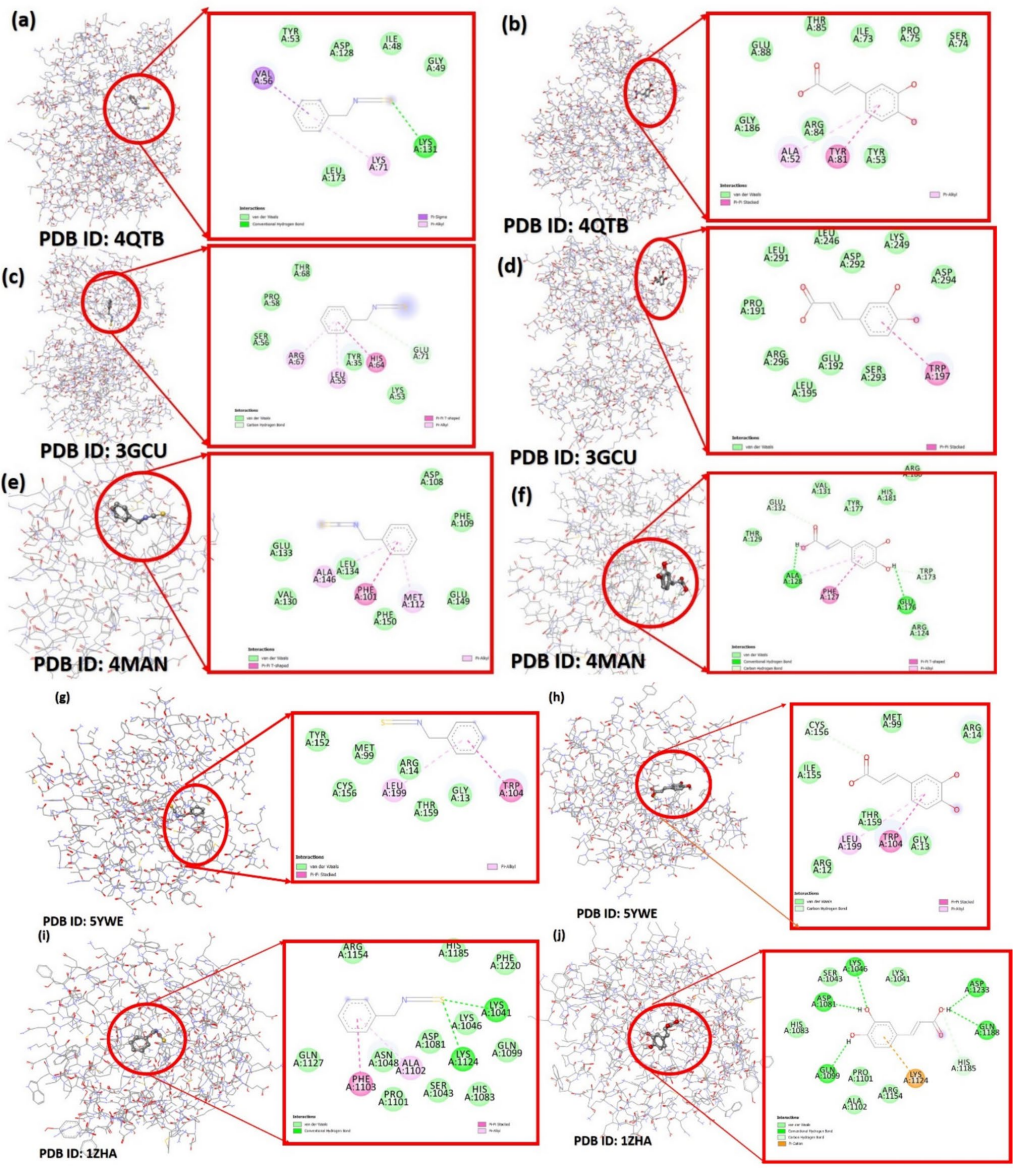
2D schematic representation of the binding interactions between benzyl isothiocyanate (BITC) and caffeic acid (CA) with key protein targets. The panels depict the binding modes to: **a**, **b** ERK2 kinase domain (PDB ID: 4QTB), **c**, **d** p38 MAPK kinase domain (PDB ID: 3GCU), **e**, **f** Bcl-2 protein (PDB ID: 4MAN), **g**, **h** Keap1-Nrf2 complex (PDB ID: 5YWE), and **i**, **j** Glutathione S-transferase (GST) (PDB ID: 1ZHA). Different interaction types (e.g., hydrogen bonds, hydrophobic interactions) are color-coded for clarity

Molecular docking simulations were employed to identify optimal binding conformations of BITC and CA to X-ray crystal structures of key protein targets implicated in the MAPK pathway, apoptosis regulation, and oxidative stress. These pathways are known to be involved in the anticancer mechanisms of these compounds. The protein type, name, and PDB ID of the target proteins are listed in Table 2. The top-ranked docking poses of each protein with BITC and CA were selected and analyzed. The results are presented in Fig. 6.

**Table 2:** Selected receptor proteins for molecular docking studies based on their roles in the MAPK pathway, apoptosis regulation, and oxidative stress mechanisms associated with the anticancer effects of benzyl isothiocyanate (BITC) and caffeic acid (CA)

SN	Protein type	Protein name	PDB ID	Benzyl isothiocyanate kcal/mol	Caffeic acid kcal/mol
1	MAPK Pathway Proteins	ERK1/2 (Extracellular signal-regulated kinases)	4QTB (Structure of ERK2 bound to a small molecule inhibitor)	−4.9	−6.5
		p38 MAPK	3GCU (Structure of p38 MAPK with an inhibitor)	−5.3	−6.8
2	Apoptosis-Related Proteins	Bcl-2 (B-cell lymphoma 2)	4MAN (Bcl-2 protein bound to an antagonist)	−5.5	−6.2
3	Oxidative Stress and Redox Homeostasis	Nrf-2 (Nuclear factor erythroid 2-related factor 2)	5YWE (Keap1-Nrf2 complex structure)	−5.3	−6.3
		GST (Glutathione S-transferase)	1ZHA (Structure of GST with a ligand)	−4.9	−6.0

To investigate the potential binding interactions of the bioactive compounds benzyl isothiocyanate (BITC) and caffeic acid (CA) with key protein targets, molecular docking simulations were performed. The 3D structures of ERK1/2 (PDB ID: 4QTB), p38 MAPK (PDB ID: 3GCU), B-cell lymphoma-2 (Bcl-2, PDB ID: 4MAN), Nuclear factor erythroid 2-related factor 2 (Nrf2, PDB ID: 5YWE), and Glutathione S-transferase (GST, PDB ID: 1ZHA) were used as receptor targets.

The top-ranked docking poses of BITC and CA with each protein target, characterized by favorable binding energies (Table 2), were selected for further analysis. Two-dimensional (2D) representations of these binding poses were generated to visualize the specific interactions between the ligands and the amino acid residues within the binding sites (Fig. 6a–j). Different interaction types, such as hydrogen bonds and hydrophobic interactions, are highlighted in various colors.

**Fig. 7:**
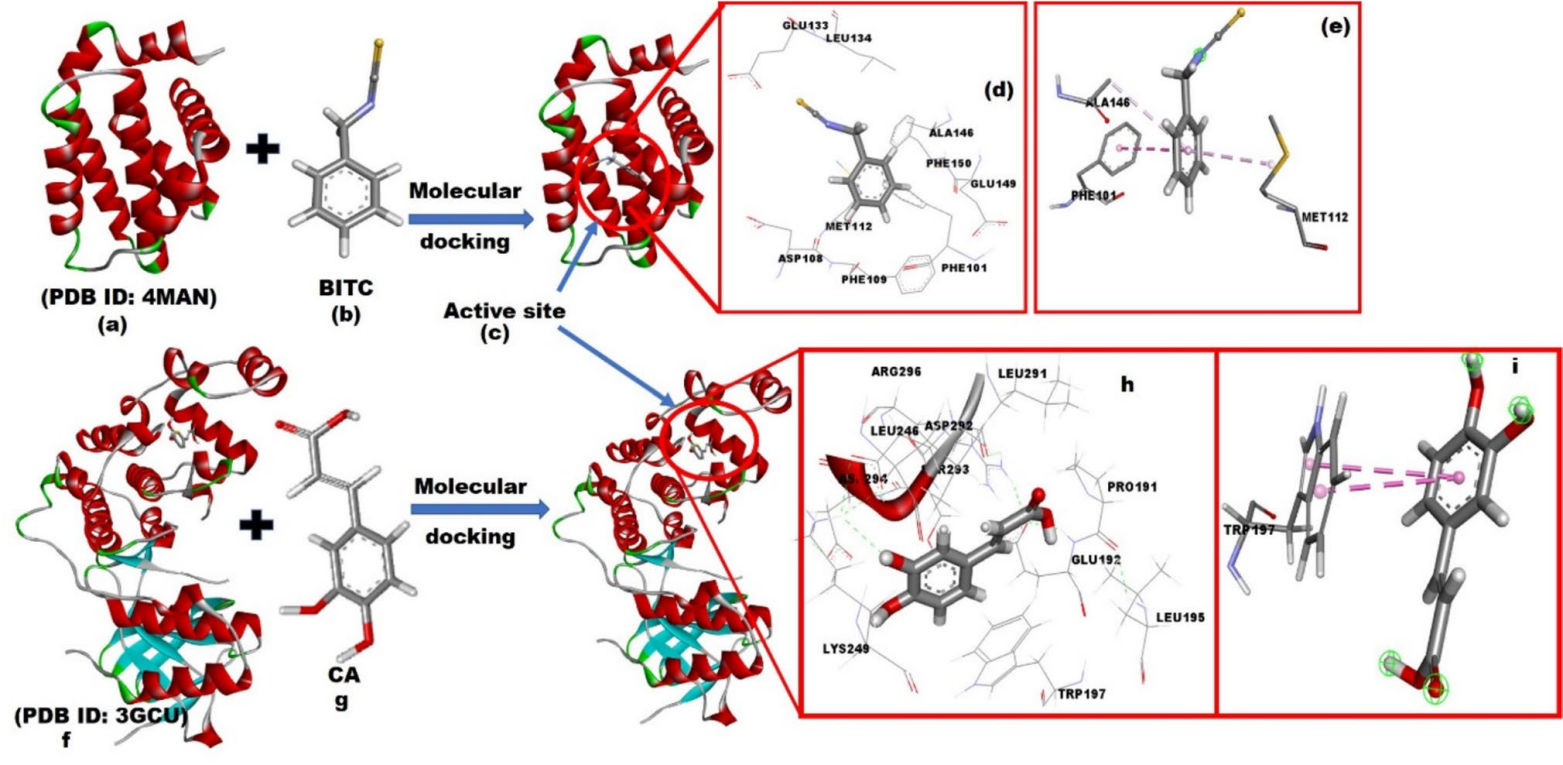
Interaction profile of receptors (**a**, **f**) PDB: 4MAN and 3GCU with ligands (**b**, **g**) BITC and CA, respectively. The active site configurations are shown in (**c**), highlighting various interactions, while (**d**, **h**) present the 3D representations of ligand-receptor binding. Ligand interactions involving π–π stacking with the receptor are depicted in (**e**, **f**)

The top-scoring poses of BITC and CA, exhibiting binding energies of −5.5 kcal/mol and −6.8 kcal/mol, respectively, were identified within the PDB structures 4MAN (Bcl-2 protein bound to an antagonist) and 3GCU (Structure of p38MAPK with an inhibitor) (Table 2). These poses were selected for further visualization and interaction analysis. The studied compounds demonstrated a variety of binding interactions with their target receptors, including hydrogen bonds, π-interactions, and van der Waals forces, as illustrated in Figs. 6 and 7. Different hues in the figures represent different types of interactions. For instance, benzyl isothiocyanate (BITC) exhibited a π-π T-shaped interaction with PHE101 and a π-alkyl interaction with ALA146 and MET112 within the active site of the 4MAN protein (Bcl-2 protein bound to an antagonist). These interactions, depicted in Fig. 7a, c, e, contributed to the stabilization of the protein–ligand complex with a binding energy of −5.5 kcal/mol. In the case of caffeic acid interactions with receptor (Fig. 7f; PDB: 3GCU), the molecular docking analysis of caffeic acid (CA) with the receptor 3GCU revealed significant interactions. As shown in Fig. 7f, CA (Fig. 7g) established a strong π–π stacking interaction between its aromatic ring and the amino acid tryptophan (TRP197) within the receptor (Fig. 7h, i). This interaction is further visualized in Figs. 6 and 7, demonstrating the spatial orientation and binding configuration. The calculated binding energy for this interaction was determined to be -6.8 kcal/mol, indicating a favorable binding affinity.

Based on docking analysis (Table 2, Figs. 6 and 7), both compounds interact closely with receptors, forming non-bonding contacts with various key amino acids, including hydrophobic and hydrophilic amino acids, to provide close contacts between receptor and ligand to form a stable complex for preventing the spread of infected cells within a living system. The overall docking results revealed that both medications can synergize for greater outcomes against cancer cell lines because they interact with a variety of critical active amino acids through secondary forces engagement in the protein–ligand complex.

**Fig. 8:**
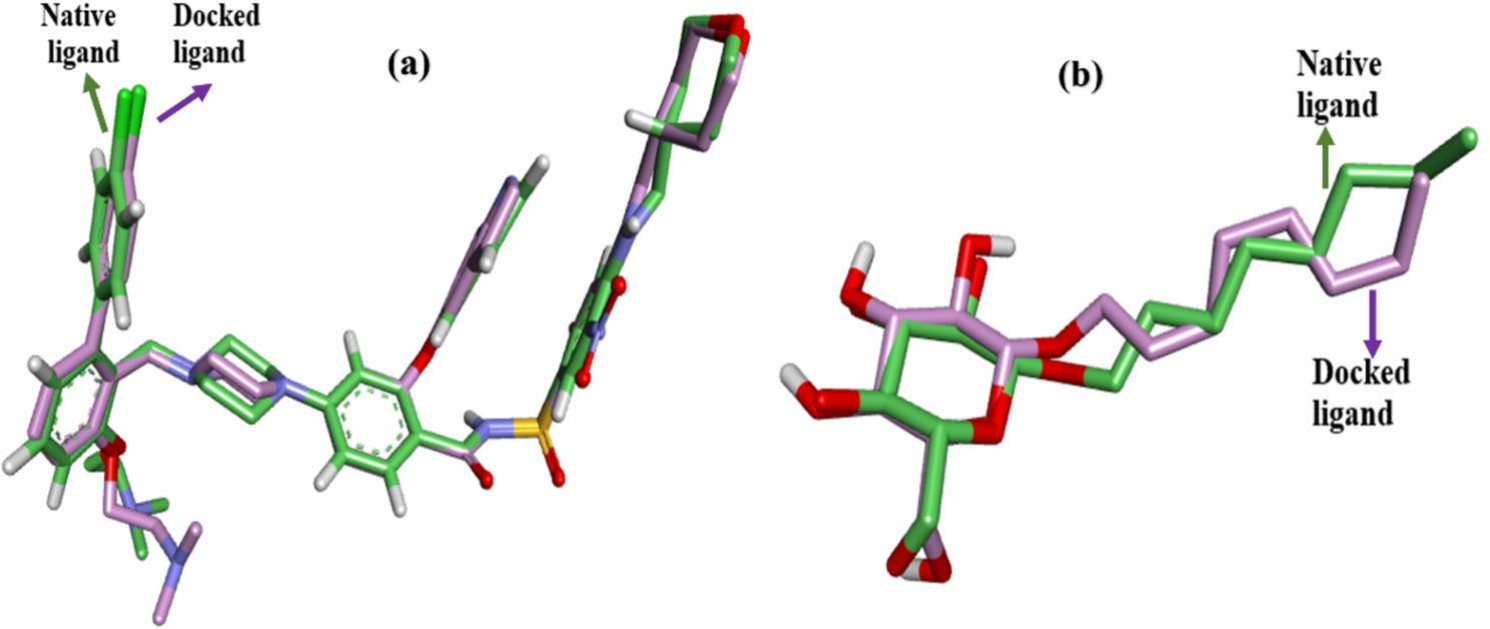
**a** Re-docking pose of the ligand in PDB 4MAN with an RMSD value of 1.129 Å (green = native ligand, purple = docked ligand). **b** Re-docking pose of the ligand in PDB 3GCU with an RMSD value of 0.871 Å (green = native ligand, purple = docked ligand)

To validate the accuracy of the molecular docking protocol, re-docking of the X-ray bound ligands into their respective active sites was performed. Figures 8a, b illustrate the superimposition of the native (green) and re-docked (purple) ligand poses for PDB 4MAN (RMSD = 1.129 Å) and PDB 3GCU (RMSD = 0.871 Å), respectively.

**Fig. 9:**
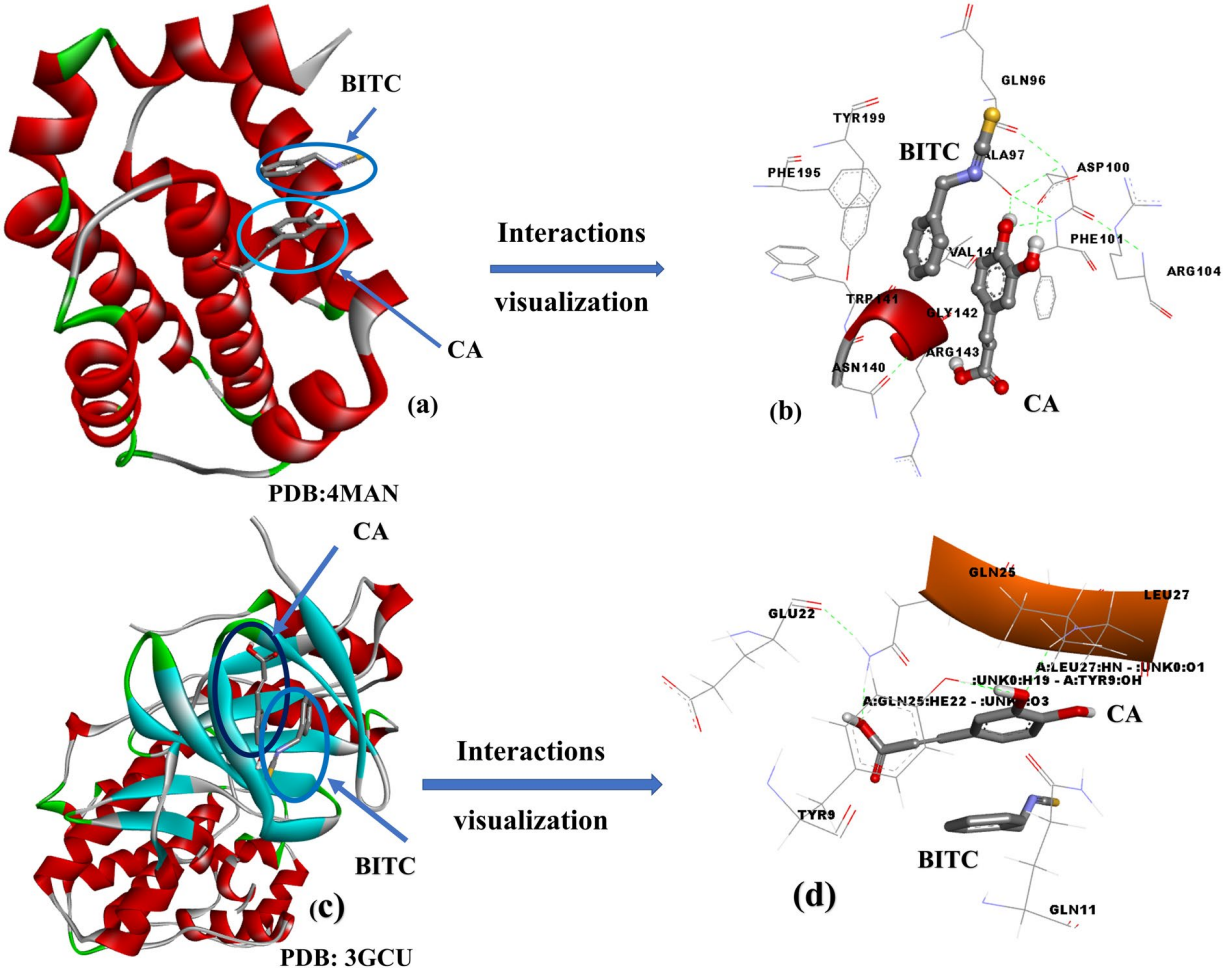
Visualization of Multiple Ligand Simultaneous Docking (MLSD) of BITC and CA with Bcl-2 (PDB: 4MAN) and p38 MAPK (PDB: 3GCU). MLSD results show enhanced binding affinities, indicating potential synergistic interactions

The top-scoring poses of BITC and CA, showing binding energies of –5.5 kcal/mol and –6.8 kcal/mol, respectively, identified within the PDB structures 4MAN (Bcl-2 protein bound to an antagonist) and 3GCU (structure of p38 MAPK with an inhibitor), were further used to evaluate potential synergistic effects through Multiple Ligand Simultaneous Docking (MLSD). In the MLSD mode, the BITC + CA pair showed enhanced binding affinities of –11.14 kcal/mol with Bcl-2 and –8.843 kcal/mol with p38 MAPK, compared to their individual docking scores (Fig. 9).

In the Bcl-2 complex (4MAN), BITC formed interactions with key residues including PHE195, TYR199, and MET112, predominantly through hydrophobic and π-π stacking interactions, while CA engaged in hydrogen bonding with ARG104, GLU136, and polar interactions with SER105. Together, the two ligands occupied adjacent yet complementary pockets, suggesting cooperative binding (Fig. 9a, b).

In the p38 MAPK complex (3GCU), BITC interacted with residues such as TYR35 and VAL38 via hydrophobic interactions, while CA formed hydrogen bonds with GLU71 and ASN155, and also engaged in π-π interaction with PHE169. The dual occupancy stabilized the active site through a network of non-covalent interactions (Fig. 9c, d).

**Fig. 10:**
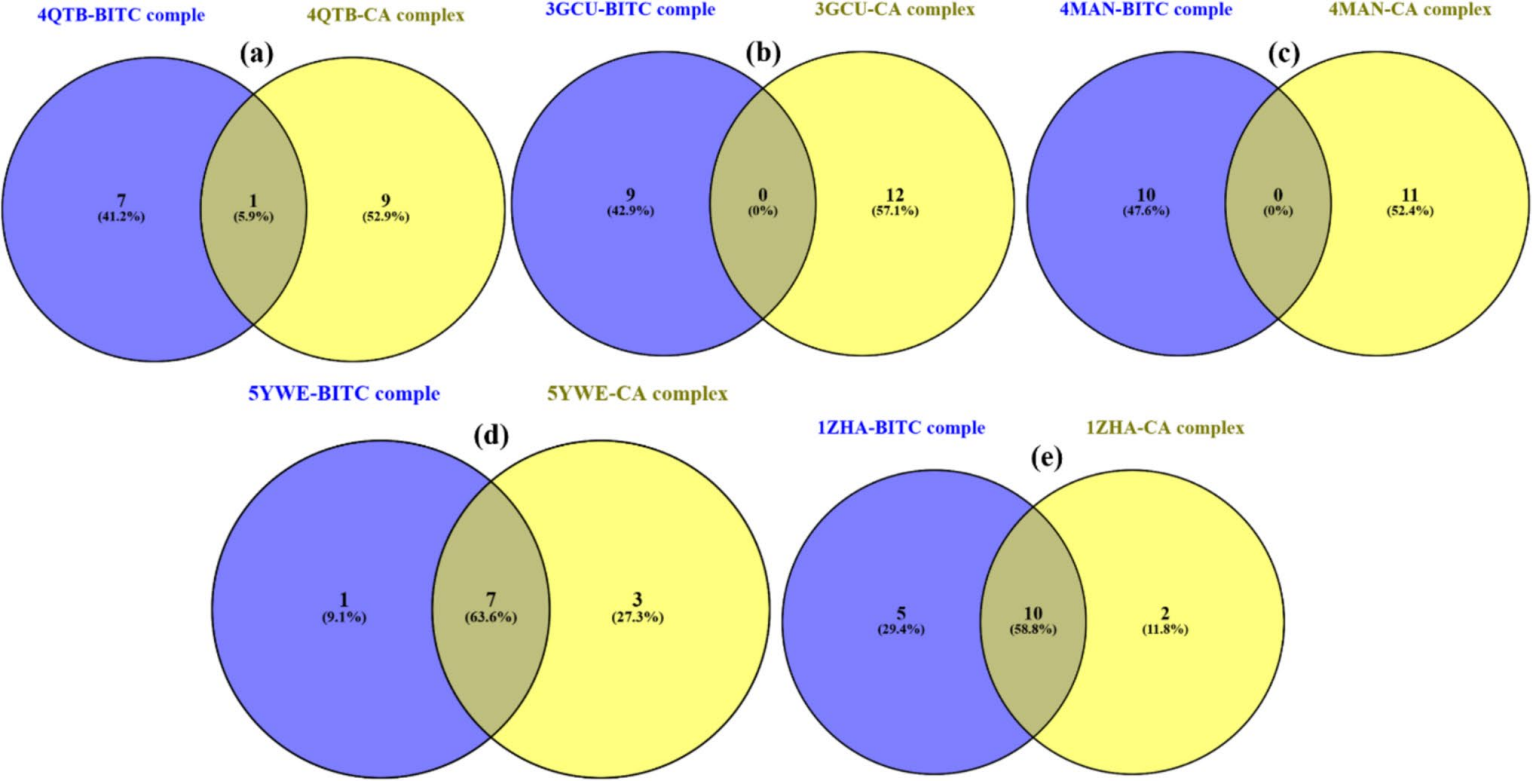
Venn diagrams showing unique and shared amino acid residues in the binding of BITC and CA to cancer-related proteins (ERK2, p38 MAPK, Bcl-2, Keap1-Nrf2, and GST), highlighting distinct and overlapping binding sites that suggest selective and synergistic mechanisms in modulating protein functions

However, to confirm these positive effects, more computational simulations and exploratory research studies are needed. Venn diagrams (Fig. 10) comparing the amino acid residues involved in the binding of BITC and CA to specific proteins (PDB entries: 4QTB, 3GCU, 4MAN, 5YWE, and 1ZHA). For the 4QTB (ERK2) complex, BITC interacted with 7 unique residues, while CA interacted with 9 unique residues, of which only 1 residue was shared in the interaction, indicating distinct but overlapping binding sites. In the 3GCU (p38 MAPK) complex, BITC interacted with 9 residues, all of which were unique, while CA interacted with 12 residues, all of which were also unique, with no overlap observed, indicating that the binding sites of the two compounds are completely different. For the 4MAN (Bcl-2) complex, BITC bound to 10 unique residues, while CA bound to 11 unique residues, with no shared residues, further confirming the distinct binding interactions. In contrast, 5YWE (Keap1-Nrf2 complex) showed that BITC interacted with 1 unique residue, CA interacted with 3 unique residues, and 7 residues were shared, which indicated a high degree of overlap, suggesting that both compounds have similar binding modes in this protein. Finally, in the 1ZHA (GST) complex, BITC bound to 5 unique residues, CA bound to 2 unique residues, and 10 residues were shared, which highlighted a high degree of overlap, suggesting that both compounds interact with similar regions in GST.

## Discussion

It's important to note that while MCF-7 cells were chosen due to their estrogen receptor positivity and well-characterized apoptotic signaling pathways (Clarke et al. 2021), further evaluation of BITC and CA combinations in other breast cancer subtypes particularly triple-negative lines such as MDA-MB-231 will be necessary to establish the broader applicability of these findings. The concentration range of BITC and CA used in this study reflects pharmacologically relevant doses. BITC plasma levels of ~ 30 µM have been reported in animal models, and CA concentrations used (10–100 µM) are within the range of physiological and dietary relevance (Ji et al. 2005; Olthof et al. 2001). Selective cytotoxicity was assessed using CRL-2522 human fibroblasts, where BITC + CA combinations showed minimal toxicity. Further validation across additional normal human cell types, such as MCF-10A, will be necessary in future studies. While this study identifies ROS generation and GSH depletion as mechanisms of cell death, confirmation of ROS-dependency using scavengers like NAC is warranted and planned for future investigations. Western blotting was used for protein expression quantification. Densitometric analyses were performed on biological triplicates and normalized to total protein levels using PVDF staining. Although MAPK pathway activation was observed, specific pathway inhibition (e.g., via SB203580 or U0126) was not performed. Such experiments are planned to further confirm the role of this pathway in BITC + CA synergy.

Previous research has shown that BITC can kill breast cancer and other cancer cells in vitro (Xiao et al. 2008; Sahu et al. 2009). On the other hand, a single treatment of CA is not an ideal candidate for breast cancer treatment as it is not potent enough to kill MCF-7 cells with unpromising cytotoxic effects, but it does suppress further proliferation of MCF-7 cells as reported previously (Jayaprakasam et al. 2006; Rajendra Prasad et al. 2011; Rosendahl et al. 2015). Surprisingly, combination treatment increased cytotoxicity toward MCF-7 cells with the increased concentration of the BITC, CA, and the time of exposure as manifested in the cell death morphologies after AO/EB dual staining. BITC induces marginal toxicity on human fibroblast cells (CRL-2522) and can be seen without reaching any EC_50_ value after 48 h, indicating less toxicity towards non-cancerous cells. All treatments were not toxic to human fibroblast cells, and we suggested this study be taken to another level of study using animal models and potentially be tested in clinical trials afterward. Furthermore, combination 4 in Table 1 possesses the second highest DRI value of BITC, which suggests 111-fold of BITC could be reduced to induce 95.25% of cell death and having a CI value of 0.009, the authors suggested this combination treatment is the best among the sets of BITC + 10 µM CA combinations. For BITC + 100 µM CA sets of treatments, the authors suggested combination 1 (48 h) displayed the best synergism with the lowest CI value among all 48 h treatment sets and had the highest DRI for BITC. Since 10 and 100 µM CA do not significantly reduce MCF-7 viability compared to BITC, DRI of CA is not being considered to find out the best synergism of any particular combination to induce MCF-7 cell death. In line with other studies (Xiao et al. 2008; Sahu et al. 2009; Rahaman et al. 2015), BITC elevated the generation of ROS to induce cellular oxidative stress, which activates cascades of cancer cell death signaling. In contrast, CA is a type of antioxidant that is able to neutralize cellular oxidative stress, thus lowering ROS levels. However, BITC in combination treatments failed to retain or increase ROS generation as a result of the CA-ROS neutralization reaction, yet still produced fascinating synergistic effects to induce MCF-7 cell death. Nrf2 signaling, together with its inhibitor, Kelch-like ECH-associated protein 1 (KEAP1) is the core of cellular antioxidant capability located upstream of the oxidative stress pathway. It is tightly regulated in determining cell fate by stabilizing the thiol proteome and regulating the expression of genes involved in response to cellular stress. Upregulation of Nrf2 causes it to bind and activate antioxidant responsive elements (AREs)-related genes and regulates response to overcome cellular stress. The significant upregulation of Nrf2 by BITC + CA combination treatments reduces ROS levels by the capability of Nrf2 signaling to directly control various cryoprotective phase II xenobiotic enzymatic and non-enzymatic cellular antioxidants. Tight regulation of Nrf2 can be seen by the down-regulation of Nrf2 after 48 h of treatment to prevent further cryoprotective mechanisms by Nrf2. Up-regulation of Nrf2 after 24 h is beneficial but not for 48 h treatment because further cell protection by Nrf2 might protect and drive the cancer cells to be resistant to further BITC + CA treatment. Down-regulation of Nrf2 reduces subsequent activation of phase II enzymes as a guardian to protect the cell. If Nrf2 is upregulated after 48 h, Nrf2 might convert its cellular stress response into an adaptive and cryoprotective response that might explain molecular insights of cancer evolution toward resistance. ROS levels were measured using DCFDA and GSH using OPA—both widely validated fluorescent probes. The inclusion of appropriate positive controls further supported the specificity and reliability of these assays. Future work may explore additional redox indicators, such as GSH/GSSG ratios or specific redox enzyme activities (e.g., glutathione peroxidase), for deeper mechanistic insights. In the context of cancer, NF-κB signaling might protect the cells from apoptosis by inducing the activation of anti-apoptotic proteins such as Bcl-2, IAP-1, and -2 and promote their growth by regulating components in cell cycle machinery (Perkins 2004; Aggarwal 2004; Zhang et al. 2017). Suppression of NF-κB expression by BITC inhibits the growth of MCF-7, limiting further activation of pro-survival signaling.

The MAPK pathway is well documented for its involvement in cell survival and death (Roberts and Der 2007; Dhillon et al. 2007). BITC activates MAPK family molecules via the generation of ROS (Xiao et al. 2008; Sahu et al. 2009) but induction of MAPK activation via the combination of BITC + CA treatment on cancer cells remains unclear as ROS level was depleted. Therefore, we discovered the role of the MAPK pathway and found that BITC + CA mediated pro-apoptotic signaling via p38 MAPK activation while suppressing pro-survival signaling via ERK1/2 and ERK phosphorylation (Tentative mechanism in Fig. 10). Suppressing ERK activations support the anticancer effect in this study as up-regulation of ERK may contribute to cancer cell survival and progression to promote cancer growth (Roberts and Der 2007). Single treatment of BITC exerts suppression of ERK (Ho et al. 2011) causing the significant suppression expression on ERK phosphorylation also influenced significant ERK depletion in BITC + CA combination treatment on MCF-7 cells at 24 h. Moreover, ERK1 and ERK2 MAPK contribute to critical roles in the regulation of cell survival and proliferation, particularly caused by mutational activation of upstream signaling components that activate ERK MAPKs (Roberts and Der 2007). Here we demonstrated the capability of bioactive compound combinations from edible sources to perform dietary synergism in down-regulating ERK signaling to boost the death of breast cancer cells in vitro. Hence, suppression of ERK by BITC + CA might be the essential finding in this study as this quality has stimulated intensive efforts by the pharmaceutical industry and research community to develop novel inhibitors that specifically block and inactivate ERK protein for cancer treatment (Roberts and Der 2007). MAPK3 (encoded for ERK1 protein) and MAPK1 (encode for ERK2 protein) have crucial roles in cellular differentiation, proliferation, and survival. MAPK3 gene expression was consistent with the translation dogma as ERK1 protein was upregulated after BITC treatment at 24 h. Beyond the pathways investigated in this study, the observed synergy may also involve other signaling cascades, such as PI3K/Akt and p53, which warrant further exploration in follow-up studies.

**Fig. 11:**
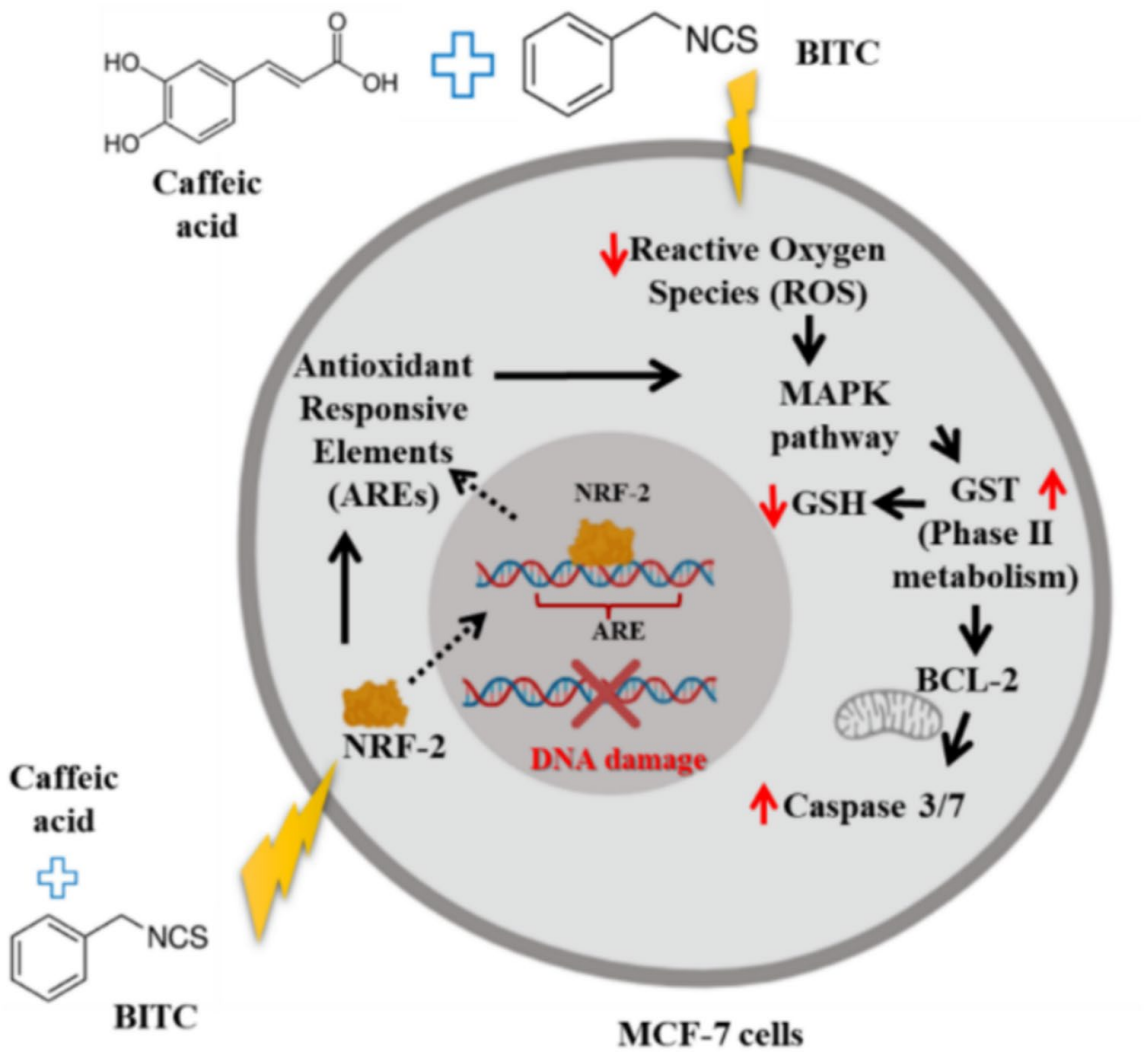
Depicts a possible mechanism by which BITC and CA together caused MCF-7 cell death

GST is involved in phase II xenobiotic metabolism that catalyzes the conjugation of glutathione (GSH) with BITC for detoxification and secretion in conjunction with the mercapturic pathway (Nakamura et al. 2000; Rahaman et al. 2018; Tan and Spivack 2009). BITC has been reported to enhance the GST activity and further synthesis of GST in treated cells (Nakamura et al. 2000). GSH is a tripeptide (l- glutamyl-l-cysteinyl-glycine) that functions to maintain redox homeostasis to protect cells from free radical damage. Depletion of GSH level after exposure to xenobiotic chemicals is one of the characteristics of cell stress that will lead to death (Circu and Yee Aw 2008). In addition, the ROS generated by a single treatment of BITC is overcome by the oxidation of GSH to oxidized glutathione (GSSG) in the reaction catalyzed by glutathione peroxidase (GP_x_) and GST, resulting in H_2_O_2_ being converted into water (H_2_O). As GSH is considered to be the major thiol-disulfide redox buffer of the cell, the measurement of GSH was used as an indicator of the state of the cellular redox environment of MCF-7 cells (Schafer and Buettner 2001). Since BITC + CA is toxic to MCF-7 cells, Nrf2 caused more free GSH to be made in the cytoplasm so that it could be attached to BITC and CA. GST then acted as a catalyst to get rid of BITC + CA from the intracellular environment. Based on our findings, CA seems to influence intense GSH depletion compared to BITC, in line with a previous study by Prasad et al. that shows significant depletion of GSH levels treated with CA (Rajendra Prasad et al. 2011). We speculate that further conjugation of BITC and CA with GSH for detoxification purposes will disrupt thiol proteome and cellular redox homeostasis as it will result in less free-GSH available to protect the cells from other GSH-unconjugated xenobiotics and oxidative stress. Bcl-2 family proteins are known to influence mitochondrial membrane stability and control the release of cytochrome c in the regulation of apoptosis. Bcl-2 family is divided into two subgroups which are anti-apoptotic (Bcl-2, Bcl-X_L_, Bcl-W, Mcl-1, and A1) and pro-apoptotic that further divided into the Bax family (Bax, Bak, and Bok) and BH-3 only family (Bid, Bim, Bik, Bad, Bmf, Hrk, Noxa, and Puma) (Cory and Adams 2002). In contrast with this, Bcl-2 was up-regulated after the treatment with BITC, CA, and BITC + CA. Articulation from previous studies (Van Delft et al. 2006; Van Delft and Huang 2006), we speculated that up-regulation of Bcl-2 after BITC + CA treatment is the counter-response of treated MCF-7 cells because of the increment of other pro-apoptotic Bcl-2 proteins and also the reduction of BH3 protein. Moreover, resisting cell death is one of the iconic hallmarks of cancer reviewed by Hanahan and Weinberg, alternatively by up-regulating the expression of anti-apoptotic regulators such as BCL-2 and BCL-X_L_ while down-regulating pro-apoptotic factors such as Bax, Bim, and Puma (Hanahan and Weinberg 2011). We suggested that this is one of the survival strategies, as by increasing the expression of pro-survival Bcl-2, the release of cytochrome c will be decreased. However, previously permanent mitochondrial membrane disruption irreversibly releases cytochrome c that activates caspase 3/7 and will continue further the cascades of cell death signaling, thus making the death process unstoppable even though the Bcl-2 increment strategy by MCF-7 cells could be seen. We also suggested the increment of Bcl-2 expression is probably a response of treated MCF-7 cells to the increased level of caspase 3/7 associated with mitochondrial disruption. Our analysis indicated that disruption of mitochondrial transmembrane potential (ΔΨ_m_) activity of MCF-7 cells after the exposure of BITC, CA, and their combinations affect the integrity of the mitochondrial membrane, thus forming pores that release cytochrome c that eventually initiate cascades of cell death mechanisms. We suggest that higher retention of Rh123 at 1 and 2 h of treatments associated with upregulation of Bcl-2 anti-apoptotic protein that protects mitochondrial membrane integrity by inhibiting the function of pro-apoptotic protein such as Bax through the formation of Bax-Bcl-2 heterodimer (El-Deiry 1998). However, the integrity of the mitochondrial membrane was disrupted starting from 4 until 24 h for both single and combination treatments, suggesting the formation of pores by the pro-apoptotic Bcl-2 protein family. Zhang et al. (2016) described the details of pore formation on the mitochondrial outer membrane, which is initiated by BH3-in-groove dimers that embed shallowly in the leaflet of the mitochondrial lipid bilayer to increase membrane tension, thereby inducing lipid pore formation_41_, which causes cytochrome c release and caspase family activation. It is clear that caspase 3/7 is one of the apoptosis biomarkers caused by the release of cytochrome c from the mitochondrial membrane that activates cascades of caspase (Cory and Adams 2002). One of the reasons for synergism between the treatments moving forward on the death processes of treated MCF-7 cells is increased caspase 3/7 levels. Figure 11 shows a diagram of how the BITC + CA combination treatment caused MCF-7 cell death. When BITC and CA are combined, they reduce ROS while also controlling the MAPK pathway. Simultaneously, BITC + CA increased the expression and translocation of Nrf2 into the nucleus and led to the up-regulation of AREs. Regulation of AREs by Nrf2 led to a decrease of ROS at the upstream pathway and influence the activity of detoxifying enzyme GST and thiol proteome such as GSH. Regarding their pharmacokinetics, BITC has been reported to reach plasma levels of approximately 20–30 µM in rodents. While caffeic acid (CA) typically exhibits lower bioavailability due to rapid metabolism, both compounds are known to possess acceptable safety profiles. Their established metabolic stability in biological systems further supports consideration for their combined therapeutic use.

BITC + CA combination treatments boosted MCF-7 cell death. Whereas all single (BITC and CA alone) and combination compounds (BITC + CA) have slight cytotoxicity effects on human fibroblast cells and did not reach EC_50_ values after 24 and 48 h treatments. This finding indicates that the chemicals used in this study have selectivity cytotoxicity effects and might be safe for normal human cells. Moreover, our results showed the fascination of a very strong synergism relationship based on the combination index (CI) value > 0.1. Based on Fig. 6, the combination treatment between BITC and CA indicated the activation of antioxidant-responsive elements (AREs) by the upregulation of Nrf2 protein expression, thus activating other phases II antioxidant elements such as the intense expression of glutathione S-transferase (GST) and depletion of glutathione (GSH) level. Further, we found that BITC generated reactive oxygen species (ROS) and utilized the stress condition to induce MCF-7 cell death in line with the study as documented but in contrast, the combination treatment depletes ROS level and causes the process of cell death more efficiently. Both single and combination treatments activate the MAPK signaling pathway via the regulation of p38 and extracellular signal-regulated kinases (ERKs). In addition, upregulation of expression of anti-apoptotic B-cell lymphoma 2 (Bcl-2) is a counter-response to suppress the release of cytochrome c, thus activating cascades of caspases leading to a significant increment of caspase 3/7 level to further the process of apoptosis.

Our results suggest a clear interplay between oxidative stress, MAPK pathway activation, and apoptosis induction. Specifically, the observed generation of reactive oxygen species (ROS) by BITC appears to be a key upstream signal, which is significantly affected by the presence of CA. Increased ROS is often a key initiator of cellular stress responses, including activation of the MAPK cascade. The differential regulation of p38 MAPK and p-ERK/ERK1/2 phosphorylation observed in this study highlights how the BITC + CA combination triggers these pathways. Downstream of these MAPK events, we observed significant changes in Bcl-2 expression and robust activation of caspase-3/7. Given that Bcl-2 is a key anti-apoptotic protein and caspases are central executioners of programmed cell death, their regulation directly supports the observed synergistic induction of apoptosis. This sequence suggests an integrated mechanism in which the combination triggers oxidative stress, which subsequently activates specific MAPK signaling to propagate the stress signal and ultimately leads to the collapse of anti-apoptotic defenses and activation of apoptotic machinery, resulting in enhanced MCF-7 cell death.

The docking results of caffeic acid (CA) and benzyl isothiocyanate (BITC) with key cancer-related receptor proteins—including ERK2 (PDB: 4QTB), p38 MAPK (PDB: 3GCU), Bcl-2 (PDB: 4MAN), Keap1-Nrf2 complex (PDB: 5YWE), and GST (PDB: 1ZHA)—revealed strong binding interactions. These findings suggest the formation of stable ligand–receptor complexes, underscoring the potential of CA and BITC to effectively target multiple signaling proteins and contribute to their observed anticancer activity against the MCF-7 breast cancer cell line.

The amino acid residues obtained from the docking analyses, visualized using a Venny plot (Fig. 8), demonstrate both distinct and overlapping binding modes of BITC and CA. This suggests complementary mechanisms of action. For instance, in the p38 MAPK (3GCU) and Bcl-2 (4MAN) complexes, BITC and CA bound to entirely different residues—such as THR A:68 for BITC and GLU A:132 for CA—indicating spatially distinct interactions. This non-overlapping binding pattern could allow simultaneous modulation of different regions of the protein, possibly leading to enhanced inhibition of their biological functions.

In contrast, within the Keap1-Nrf2 complex (5YWE) and GST (1ZHA), both compounds shared several key binding residues. For example, in GST, both BITC and CA interacted with residues like CYS A:156 and ARG A:14. This significant overlap suggests a synergistic mechanism in modulating oxidative stress pathways, as both ligands may jointly disrupt redox homeostasis in cancer cells. A molecular docking protocol is considered reliable if the root-mean-square deviation (RMSD) between the native and docked ligand poses is less than 2 Å. The re-docking RMSD values obtained for PDB 4MAN (1.129 Å) and PDB 3GCU (0.871 Å) are well below this threshold, thus demonstrating the reliability of the molecular docking protocol employed. These two protein systems were notably chosen for validation as they also yielded the best docking scores with BITC and CA, respectively.

The Multiple Ligand Simultaneous Docking (MLSD) analysis provided compelling evidence of cooperative binding between BITC and CA to shared or distinct residues within the active sites of target proteins such as Bcl-2 and p38 MAPK. This observed cooperative interaction, characterized by enhanced binding affinity, suggests a more profound influence on protein function than could be achieved by either compound alone. Such a synergistic binding profile could potentially arise through several mechanisms: either through allosteric modulation, where the binding of one ligand at a distinct site induces conformational changes that optimize the binding affinity or efficacy of the second ligand at another site; or through dual-site interactions, where both ligands simultaneously engage different sub-regions of the same binding pocket, leading to a combined effect. For instance, for Bcl-2, the proximity of BITC and CA binding sites in our MLSD model suggests a potential for joint occlusion of a crucial interaction interface, thereby enhancing its inhibitory effect. This integrated binding strategy provides a molecular basis for the enhanced cellular effects observed, including the amplified induction of apoptosis and disruption of redox homeostasis in MCF-7 cells, as it allows for a more comprehensive or sustained inhibition of key oncogenic pathways.

When docked together into Bcl-2, the combined binding energy reached –11.14 kcal/mol, significantly stronger than individual docking scores (–5.5 and –6.2 kcal/mol, respectively). In this case, BITC occupied the hydrophobic cleft (interacting with PHE195, TYR199, and MET112), while CA formed hydrogen bonds with ARG104, GLU136, and SER105. Such complementary positioning supports the notion of cooperative inhibition, potentially enhancing pro-apoptotic signaling by interfering with Bcl-2’s regulatory role.

A similar synergistic effect was observed in the p38 MAPK protein, where BITC and CA, when docked simultaneously, exhibited a combined binding energy of –8.843 kcal/mol—again, surpassing their individual binding scores. BITC mainly contributed hydrophobic interactions with residues like TYR35 and VAL38, whereas CA engaged in hydrogen bonding with GLU71 and ASN155, along with π-π stacking with PHE169. This spatial complementarity may reinforce kinase inhibition, thereby attenuating inflammatory signaling pathways associated with cancer progression.

Interestingly, the ERK2 complex (4QTB) showed partial residue overlap between the two ligands. BITC and CA both interacted with TYR A:53 but also established unique contacts such as THR A:85 and ILE A:73, respectively, indicating a potential for both additive and synergistic effects through adjacent or partially shared binding domains. These interactions could potentially influence ERK2-mediated survival and proliferation signaling cascades in cancer cells. Improved binding energies from docking are complemented by observed functional effects in vitro, including protein expression changes (p-ERK, p38, Bcl-2), ROS elevation, and caspase activation, supporting their inhibitory roles.

The convergence of our computational findings with the cellular phenotypic outcomes is striking. The identification of stable binding interactions and particularly the cooperative binding revealed by MLSD for BITC and CA with proteins like ERK2, p38 MAPK, Bcl-2, Keap1-Nrf2, and GST provides a plausible molecular framework for the observed synergistic cytotoxicity. The ability of the combination to modulate key pathways, such as activating MAPK and inducing apoptosis, along with disrupting redox homeostasis, is likely potentiated by these direct molecular interactions, suggesting that the synergistic effects are driven by concurrent targeting of multiple, interconnected cancer-related mechanisms.

Taken together, the unique and shared interaction profiles of BITC and CA across different protein targets suggest that their combination may exert enhanced anticancer activity through multi-pathway modulation. This includes interference with cell survival (ERK2, Bcl-2), inflammation (p38 MAPK), and redox balance (Nrf2 and GST). Compared to pairs like curcumin-resveratrol (Du et al. 2013; Mohapatra et al. 2015) or epigallocatechin gallate (EGCG)-quercetin (Liu et al. 2021; Li et al. 2009), the BITC + CA combination achieves synergy at lower doses, with mechanisms targeting both oxidative stress and apoptosis, thus offering unique promise for dietary-based cancer strategies. These findings support further investigation into phytochemical-based combination therapies and underscore the need for experimental validation in vitro and in vivo to confirm the predicted synergistic effects. Although this study provides convincing evidence for the synergistic anticancer effects of BITC and CA and elucidates the molecular mechanisms, several limitations remain. Our mechanistic studies were mainly conducted in a single breast cancer cell line (MCF-7), and therefore, whether these findings are applicable to other cancer types or patient-derived models requires further validation. In addition, the efficacy and safety of this combination in biological systems remain to be determined due to the lack of in vivo studies. Interactions with standard chemotherapeutic drugs (e.g., cisplatin or doxorubicin) have not been tested, but it is reasonable to evaluate their additive or antagonistic effects in the context of translational medicine as a next step. Although MLSD simulations implemented using AutoDock Vina can predict the synergistic binding of BITC and CA, it is generally believed that direct biophysical validation (e.g., isothermal titration calorimetry [ITC] or surface plasmon resonance [SPR]) is essential for future experimental validation of these predicted interactions. Future studies will focus on extending these studies to different cancer cell lines and in vivo models and employing advanced biophysical methods to fully elucidate the allosteric or two-site interaction mechanisms driving the observed synergy.

## Materials and Methods

### Materials

MCF-7 cells (American Type Culture Collection, ATCC, USA), human fibroblast cells (CRL-2522, ATCC, USA), RPMI 1640 media (Gibco, USA), BITC (252492, Sigma-Aldrich, USA), CA (C0625, Sigma-Aldrich, USA), cisplatin (sc-200896, Santa Cruz, USA), MTT (475989, Sigma-Aldrich, USA), acridine orange (A6014, Sigma-Aldrich, USA), ethidium bromide (E1510, Sigma-Aldrich, USA), phosphate buffer saline (PBS) (79382, Sigma-Aldrich, USA) 2,7-dichlorofluorescein diacetate (DCFDA) (D6883, Sigma-Aldrich, Israel) dimethyl sulfoxide (DMSO) (Merck, Germany) and ultrapure water from water purification system Milli-Q Integral system. All other chemicals, unless stated otherwise, were obtained from Sigma-Aldrich.

### Cell culture

MCF-7 cells were maintained in RPMI-1640 medium (Gibco, USA) supplemented with 10% fetal bovine serum (FBS) and 1% penicillin–streptomycin (Gibco, USA). Human fibroblast cells were cultured in Eagle’s Minimum Essential Medium (EMEM; ATCC, USA) supplemented with 10% FBS and 1% penicillin–streptomycin. All cells were incubated at 37 °C in a humidified atmosphere containing 5% CO₂.

### Cell treatments and MTT Assay

The cells were subcultured at least three times before proceeding with the treatments. MCF-7 cells (5 × 10^3^) and human fibroblast cells (2 × 10^3^) were seeded in 96-well plates in 100 µL of complete medium and incubated overnight at 37 °C. BITC, CA, and cisplatin were dissolved in DMSO, with a final DMSO concentration of 0.01% in the culture system. The concentrations for BITC (0.1–1000 µM) and CA (10 µM and 100 µM) were selected based on preliminary dose–response experiments and reported pharmacological relevance from prior literature (Olthof et al. 2001; Xiao et al. 2006). These concentrations reflect achievable plasma levels and biological activity. Cell viability was assessed using the MTT assay, as described by Rahaman et al. (2018), with slight modifications. After 24 and 48 h of treatment, media were carefully removed and replaced with 50 µL of MTT solution (0.05 mg/mL in PBS), and the cells were incubated for 4 h at 37 °C. The resulting formazan crystals were dissolved in 100 µL of DMSO, and absorbance was measured at 570 nm using a microplate reader (Bio-Rad). Background absorbance was subtracted using wells containing only media and MTT without cells. Experiments were conducted in quadruplicate, and data were expressed as percentage of control viability.

### Synergism analysis

Analysis of synergism was done using CompuSyn software as described by Chou and Martin (2005). The combination index (CI) generated from CompuSyn software was semi-quantitatively described by Chou & Talalay (1983), where CI < 1, = 1, and > 1 indicate synergistic effect, additive effect, and antagonism, respectively. All experiments were performed at least in triplicate, and the CI and DRI values were calculated based on the average of three biological replicates. Normalized isobolograms were generated for the non-constant ratios of drug combinations, as the study was carried out by keeping constant doses of CA (10 and 100 µM) while varying BITC doses (0.1, 1, 10, 100, and 1000 µM). Synergism is indicated by CI values ranging from 0 to 1, while antagonism is indicated by CI values greater than 1. In addition to these standard CI values, Chou further refined CI classifications as: very strong synergism (< 0.1), strong synergism (0.1–0.3), synergism (0.3–0.7), moderate synergism (0.7–0.85), slight synergism (0.85–0.90), nearly additive (0.90–1.10), slight antagonism (1.10–1.20), moderate antagonism (1.20–1.45), antagonism (1.45–3.3), strong antagonism (3.3–10), and very strong antagonism (> 10). The dose-reduction index (DRI) indicates the potential fold-reduction in the dose of each drug within a synergistic range, while maintaining the same level of effectiveness, which can lead to a reduction in toxicity (Chou 2006). The DRI value is a fundamental insight to integrate this study to reduce treatment doses with the same effects, thus minimizing the toxicity to the host in animal models and clinical trials in the future.

### Acridine orange/ ethidium bromide (AO/EB) dual cell staining

MCF-7 cells were seeded in 6-well plates at a density of 1 × 10⁶ cells per well in 2 mL of complete RPMI medium and incubated overnight at 37 °C in a 5% CO₂ incubator. After attachment, the cells were treated for 24 h with 30 µM BITC, 30 µM CA, and their combinations (30 µM BITC + 10 µM CA and 30 µM BITC + 100 µM CA). AO and EB were each prepared as stock solutions in PBS (1 mg/mL), and working concentrations were freshly diluted to 2 µM AO and 6 µM EB before staining. After treatment, the media was aspirated and cells were gently washed once with PBS to remove residual compounds. Next, 100 µL of the AO/EB staining solution was added to each well and incubated for 20 min at room temperature in the dark**.** After incubation, excess stain was carefully aspirated, and cells were immediately visualized under a fluorescence inverted microscope (Olympus, Japan) using a DAPI filter. Fluorescence images were captured at 400 × magnification. Cell morphology was assessed based on standard criteria: viable cells (green), early apoptotic (green with chromatin condensation), late apoptotic (orange), and necrotic (red). Experiments were performed in triplicate, and representative images from each treatment group are shown.

### Determination of reactive oxygen species (ROS)

The ROS detection procedure followed the Kadir group (Kadir et al. 2015) with modifications. MCF-7 cells (1 × 10^5^) were seeded into 24-well plates and incubated overnight at 37 °C in a humidified 5% CO₂ incubator. On the following day, cells were treated with BITC, CA, and their combinations for defined time points (30 min to 24 h). At each time point, cells were incubated with 50 µM DCFDA (D6883, Sigma-Aldrich) diluted in serum-free RPMI-1640 for 30 min in the dark at 37 °C. Following staining, cells were washed twice with PBS and fresh media was added. Fluorescence intensity was measured using a plate reader at an excitation of 485 nm and emission of 530 nm. Values were normalized to total protein content (BCA assay). Hydrogen peroxide (0.0003%) was used as a positive control. DCFDA stock was prepared in DMSO, aliquoted, and stored at –20 °C protected from light. All measurements were performed in triplicate and results are expressed as mean ± SEM.

### Western blotting

Protein lysates were extracted from treated MCF-7 cells using RIPA buffer supplemented with protease and phosphatase inhibitors (Thermo Scientific). Protein concentration was quantified using a BCA assay, and 30 µg of total protein per sample was subjected to separation on 10% SDS-PAGE gels, as described in a previously established protocol (Rahaman et al. 2015). Following electrophoresis, proteins were transferred to PVDF membranes (Life Technologies, USA) using the Trans-Blot Turbo system (Bio-Rad, USA) for 14 min at standard settings. The membranes were activated in methanol for 1 min and washed in transfer buffer (Bjerrum Schafer-Nielsen buffer). Membranes were then blocked with 5% non-fat dry milk in TBS containing 0.1% Tween 20 (TBST) for 2 h at room temperature with gentle agitation (100 rpm). After blocking, membranes were incubated overnight at 4 °C with primary antibodies in blocking buffer, using the following dilutions: p38 MAPK (1:1000), Nrf2 (1:1000), ERK1/2 (1:1000), p-ERK (1:1000), Bcl-2 (1:1000), GST (1:600), and β-actin (1:5000). After three washes in TBST (5 min each), membranes were incubated with HRP-conjugated secondary antibodies (Goat anti-Rabbit or anti-Mouse, 1:2000) for 2 h at 4 °C. After washing with TBST (3 ×), bands were visualized using Pierce ECL Western Blotting Substrate (Thermo Scientific) and imaged using a Bio-Rad Gel Doc™ XR + system. To ensure equal loading, membranes were stained using the Pierce® Reversible Protein Stain Kit for PVDF (Thermo, USA). Densitometric analysis of the band intensities was performed and normalized to total protein using ImageLab software (Bio-Rad). Western blot experiments were independently repeated three times (n = 3 biological replicates), and representative raw data blots are shown in the supplementary information (Figure S1).

### Determination of cellular reduced glutathione of treated MCF-7 cells using O-Phthaldialdehyde

In a 24-well plate (1 mL, incubated overnight), samples were prepared and analyzed based on the protocol described by Kadir et al. with some modifications (Kadir et al. 2015)45. Pellets of treated and untreated MCF-7 cells were mixed vigorously by vortex with 200 µL of RP1 lysis buffer solution (Macherey–Nagel, USA) on ice. 200 µL of 6.5% 5-sulfosalicylic acid hydrate (SSA) (390,275, Sigma Aldrich, USA) was added to the lysate on ice for 20 min and centrifuged for 14,000 rpm (5 min, 4 °C) to precipitate protein. The supernatant fraction was diluted 10,000-fold with sodium phosphate EDTA buffer (SPEB) (0.1 M) (0.1 M sodium phosphate monobasic ReagentPlus (S0751, Sigma-Aldrich), 0.1 M sodium phosphate dibasic dehydrate (30435, Sigma-Aldrich), 0.005 M EDTA (15105-35, Nacalai tesque, Japan), pH 7.5) and 100 uL of each sample were loaded into 96-well plate. In each well, 33.33 uL of O-phthaldialdehyde (OPA) (P0532, Sigma-Aldrich, 1:50 dilution with methanol) was added and incubated for 30 min at room temperature before fluorescence reading was measured at excitation 320 nm and emission 460 nm at room temperature. Reduced L-glutathione (G4251, Sigma-Aldrich) was dissolved in the 1 mL SPEB with a final concentration of 80 μM was used to generate a standard curve for GSH quantification in the samples (data not shown). The data of all samples was normalized with control sample replicates and the individual total protein content of the diluted sample suspension using the Bradford assay.

### Determination of mitochondria membrane permeability using Rhodamine 123 (Rh123) dye

We have used Rh123 cationic fluorescent dye that permeates living cells to measure mitochondrial transmembrane potential (ΔΨm) in MCF-7 cells46 upon single and combination treatments. Rh123 is remoted by normal mitochondria, which fluoresce, and when ΔΨm is lost, the fluorescence is diminished (Tang and Zhang 2005)47. MCF-7 (1 × 104) cells were seeded in 96-well plates and equilibrated overnight in a CO2 incubator at 37 ºC. Rhodamine 123 was dissolved in DMSO and was added to each well with a final concentration of 10.2 µM in RPMI 1640 media (100 µL) containing adherent MCF-7 cells and incubated for 30 min. The cells were washed with PBS twice and replaced with fresh RMPI 1640 complete medium after the treatment was completed with a final volume of 100 µL. The fluorescence measurement was taken at 1, 2, 4, and 24 h of incubation time at an excitation of 500 nm and emission of 550 nm.

### Caspase 3/7 level determination

Caspase 3/7 levels in MCF-7 cells were determined using FAM-FLICA® Caspase Assay Kits (ImmunoChemistry Technologies, USA). MCF-7 cells were seeded in 96-well plates with 1 × 104 cells in 100 µL complete RPMI medium for each of the wells and were incubated at 37 °C in a 5% CO2 incubator overnight prior to treatments. Caspase 3/7 assay was carried out as described by the given protocol of FAM-FLICA® Caspase Assay Kits for adherent cells, and the fluorescence intensity of FAM-FLICA was measured at the excitation wavelength of 488 nm and emission wavelength of 530 nm using a fluorescence plate reader.

### Quantitativ-polymerase chain reaction (qPCR)

MCF-7 cells were seeded (1 × 105 cells) in a 24-well plate and were incubated overnight to equilibrate prior to the treatments. After 24 h treatments, cells were harvested as pellets by trypsinization and centrifugation (2000 rpm). 500 µL Trizol (Invitrogen, Life Technologies) was added to lyse the cells by homogenization and left at room temperature for 5 min to allow dissociation of nucleoprotein membranes and complexes. Total RNA was extracted from each sample according to the manufacturer’s protocol for LS Trizol reagents (Invitrogen Ltd., UK). For the synthesis of cDNA, 1 µL of 300 g random hexamer primer (Invitrogen) was added to 500 ng RNA and made up to 15 µL with RNase-free water. The mixture was heated for 5 min at 65 °C then placed on ice for 10 min. 8 µL of PCR reaction mix (1 µL of 10 µM dNTPs, 5 µL of 5 × first strand buffer, 2 µL of 0.1 DTT) and 0.5 µL Superscript II reverse transcriptase (Invitrogen, Life Technologies) were added to each sample. The samples were run for thermocycling (25 °C for 10 min, 45 °C for 90 min, and 70 °C for 15 min). The qPCR was performed using the TaqMan assay according to the manufacturer’s protocol (Thermo Fisher Scientific) and the primers used were NF-κB (Hs01042014_m1), MPAK3 (Hs00385075_m1) and GAPDH (Hs02758991_g1). GAPDH was set as the normalization loading expression.

### Molecular docking

The interaction of key cancer-related proteins, including ERK2 (PDB: 4QTB), p38 MAPK (PDB: 3GCU), Bcl-2 (PDB: 4MAN), Keap1-Nrf2 complex (PDB: 5YWE), and GST (PDB: 1ZHA) with benzyl isothiocyanate (BITC) and caffeic acid (CA) separately were studied using docking techniques with AutoDock Vina (Trott and Olson 2010) to know the binding interaction mechanism between them. For the docking study, 3D structures of receptors (Table 2) were retrieved as PDB format from RCSB PDB website. The retrieved proteins act as receptors in docking studies and were prepared to eliminate water molecules and heteroatoms to remove hindrances during docking calculation. Prior to the docking study, missing amino acids were added to the proteins, and the energy of receptors was minimized using the YASARA software (Krieger and Vriend 2014). The compounds (BITC and CA) studied in this research work were used for docking studies. These compounds were downloaded in SDF format from the PubChem website. The obtained compounds were subjected to energy minimization for a good arrangement of geometric parameters before docking. The best dock poses of both compounds with high binding energy were selected for visualization using Discovery studio (BIOVIA Discovery Studio 2016). In addition, Venn diagrams were constructed to analyze the unique and shared amino acid residues involved in the binding of benzyl isothiocyanate (BITC) and caffeic acid (CA) to cancer-related proteins, including ERK2 (4QTB), p38 MAPK (3GCU), Bcl-2 (4MAN), Keap1-Nrf2 (5YWE), and GST (1ZHA). These insights provide a structural basis for the complementary roles of BITC and CA in targeting cancer-related pathways. To validate the accuracy of the molecular docking protocol, the native co-crystallized ligands were re-docked into their respective protein active sites. The pose accuracy was assessed by computing the root-mean-square deviation (RMSD) between the re-docked and original crystallographic ligand poses (Rasyid et al. 2024). This calculation was performed using the DockRMSD for RMSD Calculation (Bell and Zhang 2019). A docking protocol is typically considered reliable if the RMSD is less than 2 Å. The re-docking RMSD values obtained consistently fell below this 2 Å threshold, thereby demonstrating the robustness of our docking methodology. Docking parameters were derived from previously validated protocols, and binding sites were corroborated by co-crystallized ligand data. Prior to docking, protein structures underwent standard preparation, encompassing energy minimization, removal of water molecules and extraneous heteroatoms, and correction of any missing residues, all to facilitate robust interaction analyses. In addition to individual docking, Multiple Ligand Simultaneous Docking (MLSD) was conducted to explore the potential synergistic binding behavior of BITC and CA when co-bound to the same protein target. Ligands and receptors were converted into.pdbqt format, and grid parameters were set to encompass the active site region. MLSD was performed using AutoDock Vina by simultaneously docking both compounds to the protein targets. The resulting co-binding conformations were assessed to identify spatial complementarity, cooperative interactions, and improved binding affinities that might not be observed during individual docking. These MLSD insights provide a deeper understanding of the synergistic potential of BITC and CA in modulating cancer-associated protein targets.

### Statistical analysis

The statistical analysis was performed using GraphPad Prism V 5.0. One-way ANOVA analysis was performed with the Dunnet post-hoc test, whereas two-way ANOVA was complimentary with the Bonferroni post-hoc test to compare the treated cells with the vehicle control. For every analysis, each value is the mean ± SEM (n = 4 or 3). * indicates P < 0.05, ** indicated P < 0.01 and *** indicated P < 0.001 significant difference between vehicle control and treated cells.

## Conclusions

In conclusion, the utilization of multiple bioactive compounds increases the dynamic and effectiveness of cancer treatment as many molecular targets are regulated. This in vitro evaluation helps elucidate the synergistic mechanism of action of BITC in combination with CA, although these findings require further investigation before translation to a clinical setting. The synergism of bioactive compounds from edible sources may serve as an adjunct to the current pipeline of cancer treatment strategies. Molecular docking studies, including Multiple Ligand Simultaneous Docking (MLSD), provided additional insight into the cooperative binding behavior of BITC and CA with cancer-related proteins. MLSD revealed that their concurrent binding enhances complex stability and supports their synergistic action at the molecular level, offering a mechanistic basis for their combined anticancer potential. These results advocate for the integration of dietary compound combinations into the evolving landscape of precision oncology. Exploiting such naturally derived synergistic interactions could strengthen existing cancer therapies, offering safer and more targeted strategies. Given the promising efficacy and established dietary safety of these compounds, advancing them into clinical development must overcome formulation challenges related to their stability and bioavailability. Future efforts should explore advanced delivery systems, such as nanoformulation or co-encapsulation strategies, to optimize their pharmacokinetic properties and enhance their efficacy and targeting. While our findings are promising, additional in vivo studies are essential to validate the anticancer efficacy, systemic safety, and pharmacological compatibility of BITC and CA combinations. We plan to extend this work in vivo using mouse models to assess pharmacokinetics, tumor inhibition, and potential toxicity profiles of BITC + CA combinations. Future clinical and preclinical investigations are crucial to harness the full potential of these bioactive dietary compounds in precision nutrition and cancer treatment.

## Supplementary Information

Below is the link to the electronic supplementary material.

Supplementary file1 (DOCX 869 KB)
