# Supplementary material for: Synergistic targeting of cancer-related proteins by benzyl isothiocyanate and caffeic acid: MLSD and cytotoxic mechanisms in MCF-7 cells
Source: 3 Biotech. 2025 Aug 11;15(9):291. doi: 10.1007/s13205-025-04469-1 (PMC12339810; doi:10.1007/s13205-025-04469-1)
Supplement: Supplementary file 1 — Supplementary file1 (DOCX 869 KB) [file 13205_2025_4469_MOESM1_ESM.docx]

Supplementary Information (SI)

Synergistic Targeting of Cancer-Related Proteins by Benzyl Isothiocyanate and Caffeic Acid: MLSD and Cytotoxic Mechanisms in MCF-7 Cells


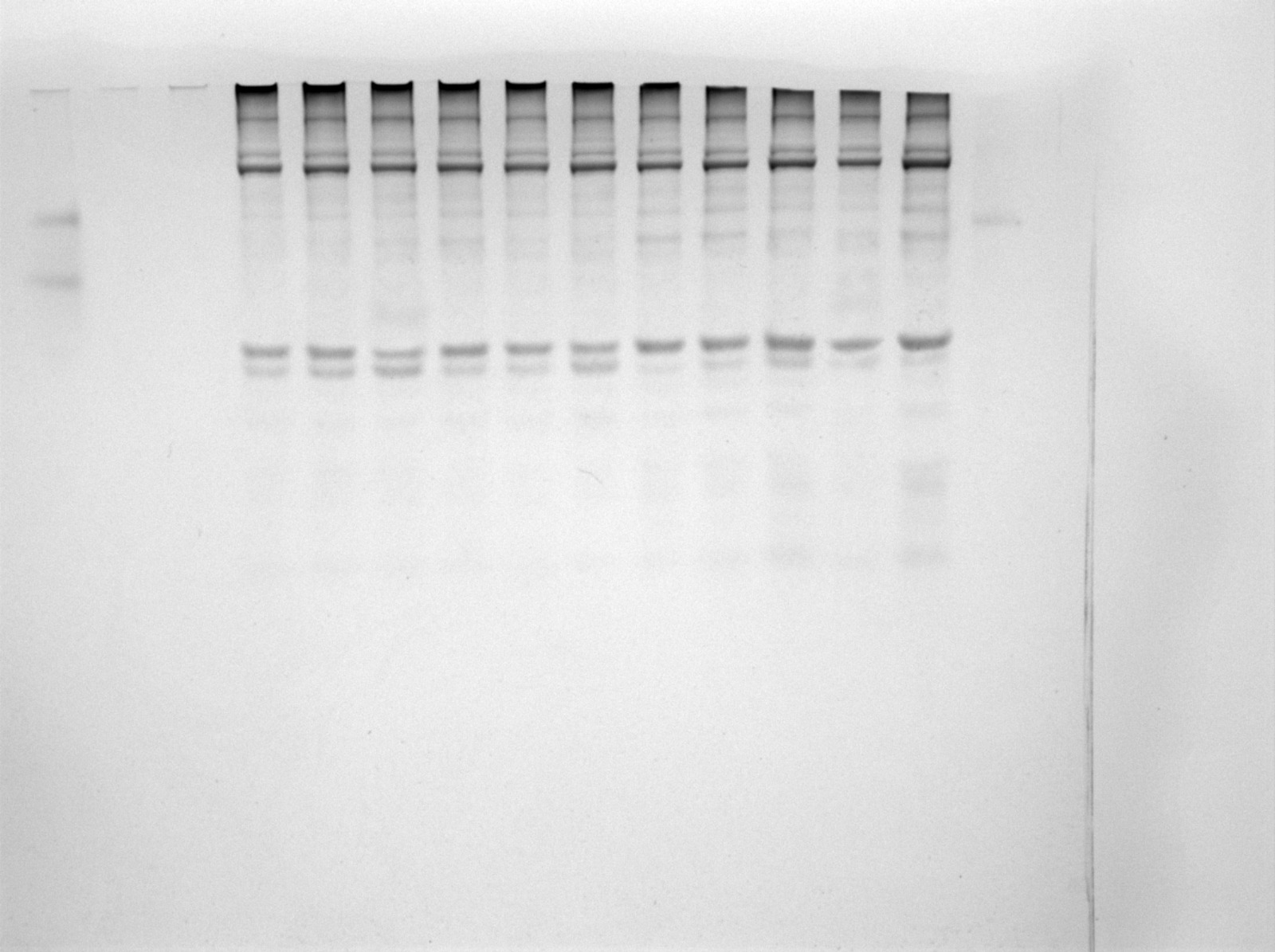


(**a**)


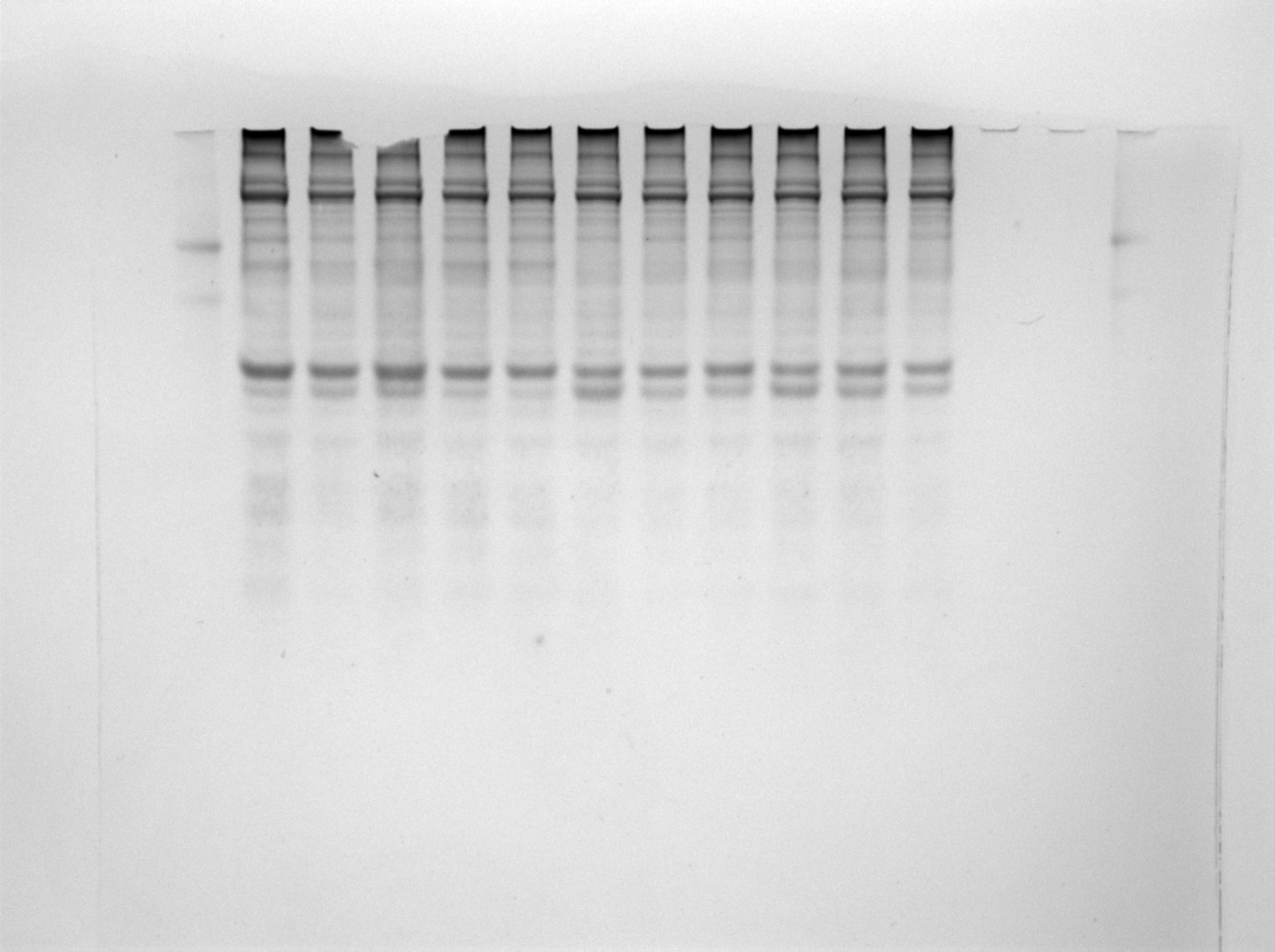


(**b**)


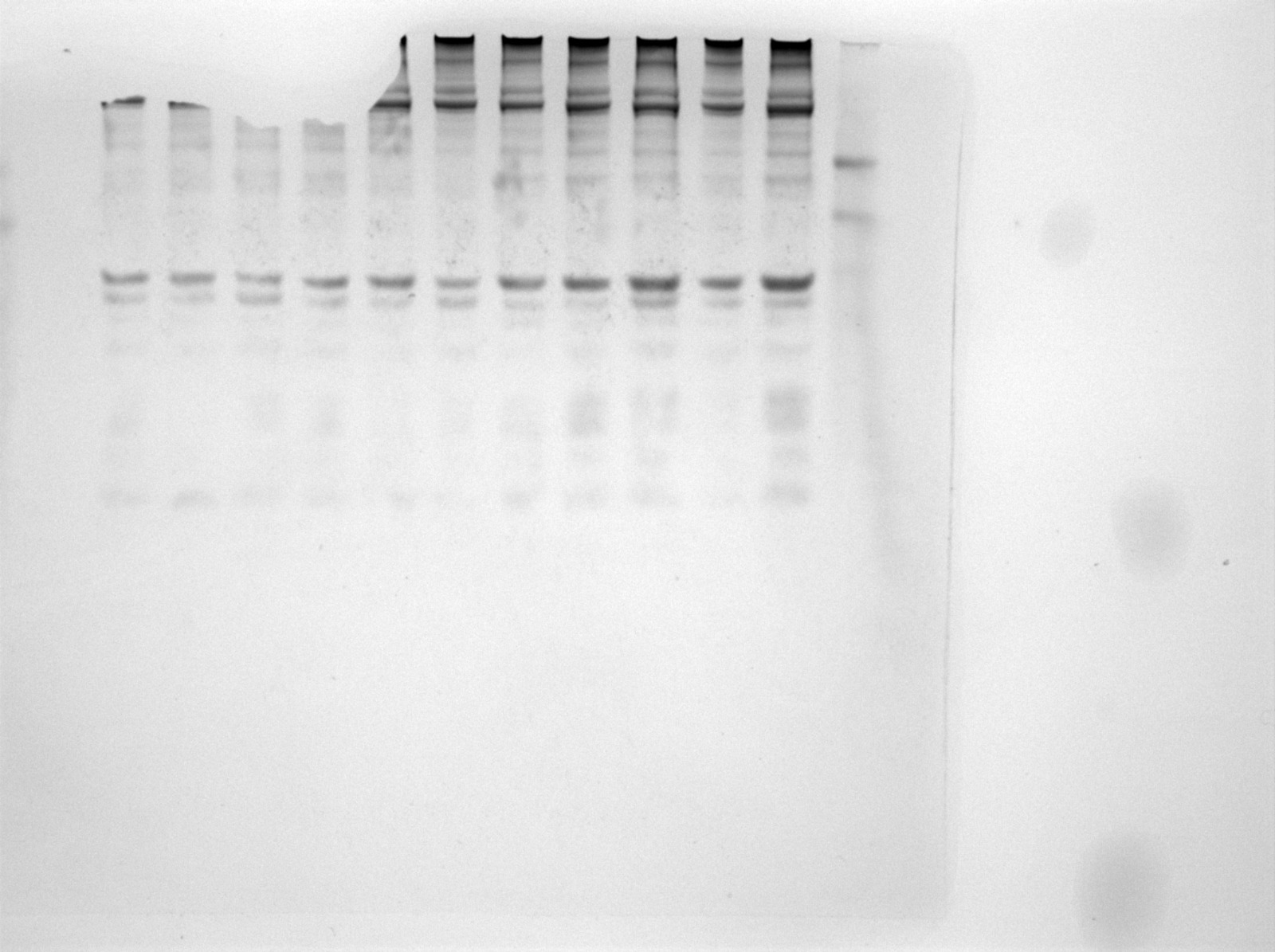


(**c**)

Figure S1. Western blot analysis of total protein expression from three biological replicates (n = 3; a, b, c) of MCF-7 cells treated with a combination of BITC and CA
